# Review of Bluetooth Low Energy RSS-Based Positioning

**DOI:** 10.3390/s26216776

**Published:** 2026-10-26

**Authors:** Fernando J. Aranda, Javier Román-Jiménez, Fernando J. Álvarez, Joaquín Torres-Sospedra

**Affiliations:** 1 Sensory Systems Research Group (GISS), Universidad de Extremadura, 06006 Badajoz, Spain; fer@unex.es (F.J.A.); jromanj@unex.es (J.R.-J.); 2 Department of Computer Science, Universitat de València, Avda. de la Universitat S/N, 46100 Burjassot, Spain; joaquin.torres@uv.es; 3 Valencian Graduate School and Research Network of Artificial Intelligence (VALGRAI), Camí de Vera S/N, Edificio 3Q, 46022 Valencia, Spain

**Keywords:** Bluetooth Low Energy (BLE), indoor positioning, Received Signal Strength (RSS), fingerprinting, proximity, model-based positioning, multi-sensor fusion, machine learning

## Abstract

Bluetooth Low Energy (BLE) Received Signal Strength (RSS)-based positioning combines low-cost deployment with widespread smartphone support. This review synthesizes peer-reviewed studies from 2013 through the search cutoff of 19 September 2026 for mobile and Internet of Things (IoT) platforms, including methods adapted from other radios and BLE-specific techniques. We systematically searched major bibliographic databases using BLE, RSS, positioning, localization, and methodological terms and then deduplicated and screened the records. The synthesis covers six dimensions: (i) RSS preprocessing and filtering; (ii) proximity-based, model-based, and fingerprinting approaches; (iii) sensor fusion; (iv) applications; (v) public BLE RSS datasets; and (vi) comparisons with other RSS-based positioning technologies. BLE RSS offers practical, cost-effective positioning in Global Navigation Satellite System (GNSS)-denied environments, but RSS instability remains the predominant limitation.

## 1. Introduction

Widely available IEEE 802.11 Wireless LAN (Wi-Fi) and Bluetooth radios support positioning and short-range communication [1]. While GNSS is standard outdoors, attenuation, multipath, and unavailable satellite Line-of-Sight (LoS) degrade indoor performance [2]. This motivates alternative Indoor Positioning System (IPS) solutions using radios that are already integrated into off-the-shelf devices.

Wi-Fi positioning benefits from existing infrastructure, but frequent scanning consumes energy, while interference, multipath, and environmental changes can limit accuracy and real-time operation on battery-powered devices. Bluetooth Classic primarily supports short-range, connection-oriented communication and is less commonly used for positioning. These limitations have encouraged lower-power alternatives such as BLE [3].

Designed for low-power IoT operation, BLE supports inexpensive deployments through smartphones and embedded devices. RSS-based methods can be implemented without specialized positioning hardware, although channel-dependent behavior, multipath, and measurement noise affect performance.

Recent BLE RSS surveys cover algorithms, challenges, and datasets for reproducible benchmarking [4], accuracy and its practical determinants [5], and fingerprinting workflows [6]. Shi and Gong provide a broader Bluetooth-localization taxonomy and compare cost, availability, scalability, and accuracy [7].

Broader reviews include a meta-review of indoor positioning surveys and Wi-Fi/BLE RSS fingerprinting [8], cross-technology trade-offs [9], technology selection for IoT applications [10], and industrial techniques, use cases, and gaps in end-to-end location services [11].

This review covers peer-reviewed BLE RSS positioning research from 2013 to 2026. It organizes proximity-based, model-based, and fingerprinting methods alongside RSS preprocessing and sensor fusion and then examines applications, public datasets, and comparisons with other RSS-based technologies.

Section 2 describes the search and eligibility criteria. Section 3, Section 4, Section 5 and Section 6 cover BLE acquisition, RSS preprocessing, positioning methods, and sensor fusion. Section 7 and Section 8 review datasets and applications; Section 9 compares technologies. Section 10 and Section 11 discuss future challenges and conclusions.

## 2. Search Criteria

### 2.1. Search Strategy and Scope

We searched Scopus (https://www.scopus.com/search/form.uri?display=advanced&zone=header&origin=, accessed on 19 September 2026), Web of Science (https://www.webofscience.com/wos/woscc/basic-search, accessed on 19 September 2026), and IEEE Xplore (https://ieeexplore.ieee.org/Xplore/home.jsp, accessed on 19 September 2026) for studies that analyze BLE RSS or explicitly use it in positioning, localization, ranging, proximity detection, or tracking.

The database searches were conducted on 19 September 2026; coverage of 2026 is limited to records available by that search cutoff.

We distinguish database retrievals, the **methodological corpus**, and contextual or supporting references. Database retrievals comprise all exported records before screening. The methodological corpus comprises the 561 journal and conference papers accepted for the final review after assessment of BLE RSS relevance and available evidence. Contextual application papers illustrate deployment domains, while supporting references provide background or documentation. Table 1 and Figure 1 describe database retrievals before final selection; they do not represent the size of the methodological corpus.

The search combined the following term groups:

- **Technology:** BLE, Bluetooth Low Energy, Bluetooth LE, iBeacon, and Eddystone.
- **Physical Quantity:** RSS, RSSI, received signal strength, and received signal strength indicator.
- **Positioning Methods:** Positioning, localization, fingerprinting, radio maps, trilateration, multilateration, distance or range estimation, and proximity detection, estimation, or classification.
- **RSS Research Topics:** Characterization, propagation, path loss, variability, filtering, preprocessing, calibration, advertising channels, channel diversity, and datasets.

This review targets English-language, peer-reviewed journal articles and full conference papers from 2013 to 2026, with database coverage of 2026 limited to 19 September 2026. Platform terms were not mandatory because they could exclude device-independent methods; smartphone, operating-system, embedded-system, and robotic-platform details were recorded during extraction.

The queries combine the technology and RSS groups with AND and the positioning and RSS-research alternatives with OR. Listings 1–3 give the database-specific syntax.

In the Scopus and Web of Science queries, an asterisk (∗) denotes truncation/wildcard matching of term variants (for example, localiz∗); the IEEE Xplore query lists the corresponding terms explicitly.

For IEEE Xplore, the 2013–2026 range and journal/conference content types were selected through the search interface. Non-English records and publications other than full peer-reviewed papers were excluded during screening.

The supplied database exports contained 3356 records: 1655 from IEEE Xplore, 723 from Web of Science, and 978 from Scopus. Deduplication used normalized DOIs and titles together with publication year and supporting metadata, with conflicting cases reviewed individually. This removed 1220 duplicate database copies and yielded 2136 unique publications: 1444 conference papers and 692 journal articles. Database rows retain the source classifications. The 2136 unique records constitute the retrieval set considered for screening, not the final set included in the methodological synthesis.

**Listing 1.** Scopus query.

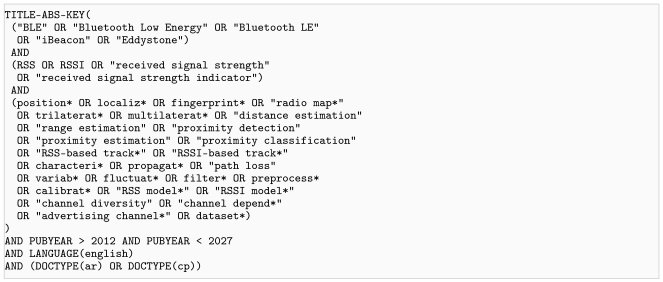



**Listing 2.** Web of Science query.

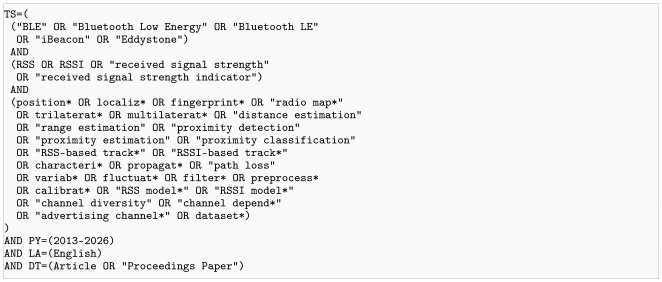



**Listing 3.** IEEE Xplore query.

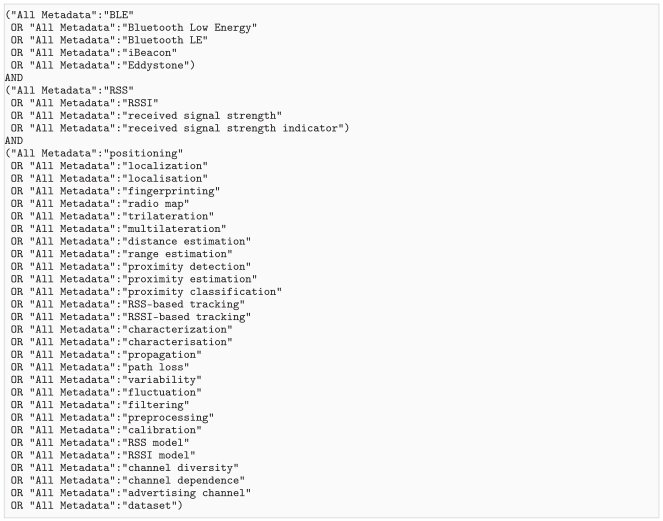



### 2.2. Eligibility and Screening Criteria

The 2136 unique database records formed the screening pool. Titles and abstracts were assessed first, and full texts were inspected when the role of BLE RSS remained unclear. Papers retained in the methodological corpus were required to satisfy the following criteria:

- **Direct Use:** Measured or simulated BLE RSS was acquired, processed, modeled, or used by the method.
- **Substantive Role:** BLE RSS contributed to the research objective, method, experimental analysis, or technical contribution.
- **Sufficient Description:** Acquisition, representation, filtering, calibration, modeling, or integration of BLE RSS was identifiable.
- **RSS-Specific Evidence:** At least one result, observation, parameter, model, or conclusion concerned BLE RSS behavior or use.
- **Publication Eligibility:** An English-language, peer-reviewed journal article or full conference paper within the review period.

Eligible topics include measurement and propagation characterization; preprocessing and calibration; proximity, ranging, model-based positioning, and fingerprinting; radio-map construction and learning; sensor fusion; hardware, antenna, channel, and deployment effects; documented public datasets; and direct comparisons with other RSS-based technologies. Multimodal studies were eligible when the BLE RSS contribution was identifiable; a separate BLE-only ablation was not required.

The following records were excluded from the core corpus:

- Publications outside the review period or eligible publication types. Reviews, standards, theses, reports, editorials, posters, and demonstration abstracts could serve as background sources.
- Studies using BLE only for communication, synchronization, identification, service discovery, or content delivery or mentioning RSS only as background or an incidental implementation detail.
- Black-box positioning services, application-only evaluations, or multimodal systems without sufficient explanation of the BLE RSS method. Application outcomes such as user satisfaction, attendance, engagement, workflow improvement, or energy savings alone did not establish methodological eligibility.
- Studies relying exclusively on time- or angle-based BLE observables, unless they also analyzed or compared an RSS-based method.

For borderline studies, the publication had to establish what BLE RSS information was used, how it entered the method, and what RSS-specific contribution or evidence was reported. Failure to establish any of these points precluded core inclusion.

From the screening records, 561 papers were accepted for the final review and are treated as a single methodological corpus. The remaining 1575 deduplicated retrieved records were not included because they did not explicitly address BLE RSS as a substantive methodological component or because the available resources did not permit full assessment and incorporation of all potentially relevant records.

The review provides a thematic synthesis rather than an exhaustive article-by-article catalogue. The discussion groups papers with related contributions rather than describing every included paper individually. Methodological tables summarize extractable characteristics and representative results, while contextual references illustrate deployment domains. Because the retrieval set was large and the available review resources were finite, the final 561-paper corpus should not be interpreted as an exhaustive set of all potentially relevant BLE RSS publications. Relevant studies may therefore exist that satisfy the broad topic scope but were not included in the final synthesis. Supporting and contextual references may also appear in the bibliography without belonging to the 561-paper methodological corpus.

### 2.3. Treatment of Application-Oriented and Supporting References

Application-focused papers were excluded from the methodological corpus when they reported only application outcomes or provided insufficient information to extract a substantive BLE RSS method, measurement finding, or evaluation result. An application study remained eligible when it independently satisfied the methodological criteria.

Selected application-only papers were cited to illustrate healthcare, navigation, safety, industry, agriculture, education, tourism, smart buildings, and crowdsensing. These examples are representative rather than exhaustive and do not contribute to methodological counts or comparisons. Any such papers retrieved by the databases remain in the all-results counts and yearly distribution.

Reviews, standards, general positioning literature, and conceptual works provide background. Dataset repository records document accessibility without counting as additional primary studies. A paper may support both methodological and application discussions, but this does not create an additional study in the corpus.

### 2.4. Classification and Data Extraction

Methodological studies could receive multiple thematic tags:

- **Application Context:** Use case or deployment domain; this tag alone did not establish eligibility.
- **Proximity:** RSS-based presence, proximity, or symbolic classification.
- **Model-Based Positioning:** Propagation-based RSS-to-distance mapping and range-based position solvers.
- **Fingerprinting:** Positioning from stored or learned RSS fingerprints.
- **RSS Modeling:** Propagation, statistical, or data-driven models for characterization, simulation, ranging, or positioning.
- **RSS Filtering:** Processing to reduce noise, outliers, instability, or temporal variability.
- **Sensor Fusion:** Integration with inertial sensors, other radios, maps, vision, or context.
- **Datasets:** Documented BLE RSS data releases and reproducibility material.
- **Other Technologies:** Direct comparisons with analogous RSS-based methods.

Where available, we extracted the environment, hardware, transmitter/receiver configuration, acquisition parameters, evaluation protocol, positioning method, performance metric, and results. Channel dependence, LoS/NLoS conditions, and PHY effects are discussed within the relevant methodological sections. Studies spanning several topics may be cited in multiple sections.

Figure 1 counts each of the 2136 deduplicated retrieved publications once, independently of methodological tags or final inclusion. Publication years follow IEEE Xplore and then Scopus, followed by Web of Science when sources disagree; original year variants remain in the merged record list. The distribution retains one Web of Science record indexed under a 2027 issue year because it was present in the 19 September 2026 export as an early-access record. Its presence in the retrieval set does not extend the core review’s 2013–2026 scope.

The search and screening cannot guarantee exhaustive coverage. Relevant publications may have been missed because of database coverage, indexing delays, terminology, language or publication-type restrictions, and the finite resources available for full-text assessment and synthesis. Screening and classification also involve judgment. Consequently, the final 561-paper corpus is a selected evidence base rather than a claim that every potentially eligible BLE RSS study was included.

## 3. Overview

### 3.1. Technology Development

Introduced in Bluetooth Core 4.0 in 2010, BLE supports short, intermittent exchanges on energy-constrained devices. Smartphone and embedded implementations make connectionless received-power measurements accessible for proximity and positioning [12].

BLE uses 40 channels spaced by 2 MHz in the 2.4 GHz Industrial, Scientific, and Medical (ISM) band (Figure 2). Channels 37, 38, and 39 carry primary advertising; channels 0–36 carry connection traffic and, since Bluetooth 5.0, secondary advertising. Multiple receivers can observe a connectionless advertiser without maintaining separate links [12].

### 3.2. RSS Measurement

RSS is a radio-produced received-power estimate exposed through a controller or platform interface. Advertising, discoverability, and connectability are distinct: Non-discoverable devices may advertise, and nonconnectable beacons provide RSS without accepting connections. Passive scanning receives advertisements; active scanning may request responses from scannable advertisers. A new conventional LE connection requires connectable advertising, even outside discoverable mode [12] (see the Bluetooth Core Specification, Volume 3, Part C, Sections 9.1–9.3).

Acquisition mode complements the algorithmic taxonomy. Connectionless observation avoids per-beacon links; connected measurements follow connection-event timing and consume communication resources. Advertising intervals, scan windows, duplicate filtering, and operating-system restrictions affect available observations. Deployment comparisons should distinguish passive scanning, active scanning, and connected sampling.

The BLE physical layer uses Gaussian frequency-shift keying (GFSK). PHY, coding, receiver sensitivity, and packet duration affect reception and sampling of received power [12] (see the Bluetooth Core Specification, Volume 6, Part A, Sections 3 and 4). Better reception does not guarantee less biased RSS-derived distances: Multipath, shadowing, and calibration remain relevant. Comparisons should document PHY and transmit power and distinguish measurement availability from positioning accuracy.

BLE legacy and Extended Advertising reports express RSSI in dBm; 127 denotes an unavailable reading. The Basic Rate/Enhanced Data Rate (BR/EDR) connected-link Host Controller Interface (HCI) command HCI_Read_RSSI instead reports a signed deviation in dB from a controller-selected receive-power range, returning zero within it. For LE connections, it reports dBm [12] (see the Bluetooth Core Specification, Volume 4, Part E, Sections 7.5.4, 7.7.65.2, and 7.7.65.13). We use RSS for BLE received-power estimates; RSSI is not inherently unitless, and the BR/EDR zero-valued interval does not apply to BLE advertising. Platform calibration, quantization, and saturation still require characterization.

## 4. RSS Filtering Techniques in BLE Positioning Systems

BLE RSS varies with propagation, devices, and advertising/scanning settings [13]. Preprocessing comprises four overlapping families: **basic techniques** (window summaries and outlier rejection); **temporal filtering** of raw or aggregated observations; **model-based recursive filtering**, including Kalman Filter (KF) and particle filters with prediction–update cycles and explicit uncertainty; and **channel-aware filtering**, which processes identified channels separately before fusion. Windows use sample counts or durations. Families can be combined: moving averages are both window summaries and temporal filters.

### 4.1. Notation

Let *r* denote an RSS measurement in dBm; unless stated otherwise, aggregation and filtering operate on these logarithmic values. The window $\left\{r_{1},r_{2},\ldots ,r_{N}\right\}$ contains samples acquired in order at a fixed position for the same transmitter–receiver pair. Alternatively, $\left\{r_{t_{1}},r_{t_{2}},\ldots ,r_{t_{\max}}\right\}$ indexes acquisition times $t_{k}$, relative to the capture start or an absolute reference, as specified. Channel- or frequency-specific values carry channel identifiers, $\left\{r^{37},r^{38},r^{39}\right\}$.

### 4.2. Basic Techniques

For a sample window $\left\{r_{1},r_{2},\ldots ,r_{N}\right\}$ collected at a fixed position for a given transmitter, the arithmetic mean, median, and maximum are as follows:

(1) $${\bar{r}}_{\mathrm{mean}}=\frac{1}{N}\sum_{i=1}^{N}r_{i},\;{\tilde{r}}_{\mathrm{median}}=\mathrm{median}\left(r_{1},\ldots ,r_{N}\right),\;{\hat{r}}_{\max}=\max\left(r_{1},\ldots ,r_{N}\right).$$

The arithmetic mean averages dBm values. Linear-power averaging gives $10\log_{10}\;\left[N^{-1}\sum_{i=1}^{N}10^{r_{i}/10}\right]$ dBm, with $10^{r_{i}/10}$ expressed numerically in mW. These summaries estimate different quantities. The median resists isolated extreme observations [14,15] but passes through additive device offsets, which require calibration or compensation. Mean smoothing reduces responsiveness to abrupt changes. The maximum can improve performance in some scenarios but is sensitive to spurious peaks, potentially from hardware rather than propagation [16]. Other variants include the mode, majority voting, and multi-level combinations of simple filters. These inexpensive techniques can reduce short-term variability relative to unfiltered signals [17].

Gaussian-based modeling and outlier detection include rejecting values more than *k* standard deviations from the mean:

(2) $$|r_{t}-\mu |>k\;\sigma \;\Rightarrow \;r_{t}\;\mathrm{discarded},$$

where μ and σ are the window mean and standard deviation, and *k* controls rejection. Ionescu et al. use a Chebyshev-based rule with k=2, followed by averaging and Kalman filtering [18]. Thresholds should reflect the measurement distribution and operating conditions.

### 4.3. Temporal (Sequential) Filtering

Temporal filtering smooths consecutive raw measurements $\left\{r_{t}\right\}$ or window summaries $\left\{\hat{r}\left[n\right]\right\}$. A moving mean is also sliding-window aggregation, so these categories overlap.

Window aggregation is as follows:

(3) $$\hat{r}\left[n\right]=g\left(\{r_{t}:\;t\in W_{n}\}\right),$$

where g(·) is an aggregation operator (e.g., mean, median, percentile) over the *n*-th time window $W_{n}$ of duration *T*. Filtering the series $\hat{r}\left[n\right]$ can reduce abrupt inter-window jumps.

A moving average filters the raw or aggregated sequence [19]:

(4) $$\tilde{r}\left[n\right]=\frac{1}{M}\sum_{k=0}^{M-1}x\left[n-k\right],\;x\left[n\right]\in \left\{r\left[n\right],\;\hat{r}\left[n\right]\right\},$$

where *M* is the filter length in samples or windows, and x[n] is the input: raw measurement r[n] or window estimate $\hat{r}\left[n\right]$.

Alternatively, Exponentially Weighted Moving Average (EWMA) uses α to balance noise reduction and responsiveness [20]:

(5) $$\tilde{r}\left[n\right]=\alpha \;x\left[n\right]+\left(1-\alpha \right)\;\tilde{r}\left[n-1\right],\;0<\alpha <1,$$

with typical initialization $\tilde{r}\left[0\right]=x\left[0\right]$. Stability comes at the cost of latency: Generally, a larger *M* or smaller α increases smoothing and effective delay. Discrete low-pass filters (e.g., Butterworth) also attenuate high-frequency RSS components, with latency determined by their temporal response [21].

### 4.4. Recursive Filters (KF and Particle Filters)

Recursive filters use dynamic models to estimate RSS, RSS-derived range, or position/navigation states at different pipeline stages. The linear scalar KF illustrates RSS preprocessing through a prediction–update cycle [22]. Its state $r_{k}$ is the underlying RSS at time step *k*. We assume a random walk, direct observations, and mutually independent, temporally white, zero-mean Gaussian process and measurement noise independent of the initial state:

(6) $$\begin{array}{cccc} \underset{\mathrm{state}}{\underbrace{r_{k}}} & =r_{k-1}+w_{k-1}, & \; & w_{k-1}∼N\left(0,Q\right),\\ \underset{\mathrm{RSS}\;\mathrm{measurement}}{\underbrace{z_{k}}} & =r_{k}+v_{k}, & \; & v_{k}∼N\left(0,R\right). \end{array}$$

(7) $$\begin{array}{cccc} \mathrm{Prediction}:\;{\hat{r}}_{k|k-1} & ={\hat{r}}_{k-1|k-1}, & P_{k|k-1} & =P_{k-1|k-1}+Q,\\ \mathrm{Gain}:\;K_{k} & =\frac{P_{k|k-1}}{P_{k|k-1}+R},\\ \mathrm{Update}:\;{\hat{r}}_{k|k} & ={\hat{r}}_{k|k-1}+K_{k}\left(z_{k}-{\hat{r}}_{k|k-1}\right), & P_{k|k} & =\left(1-K_{k}\right)\;P_{k|k-1}. \end{array}$$

where *Q*, *R*, and *P* are the process-noise, measurement-noise, and estimation-error variances, respectively, and $K_{k}$ is the Kalman gain. Typical initialization uses ${\hat{r}}_{0|0}=z_{0}$ (or an initial short average), with large $P_{0|0}$ for high initial uncertainty. The Extended Kalman Filter (EKF) and Unscented Kalman Filter (UKF) accommodate nonlinear state or observation models; changing noise statistics requires adaptive noise modeling or covariance adjustment.

Particle filters approximate state distributions with weighted particles [23]. For scalar RSS, ${\left\{r_{k}^{\left(i\right)},\omega_{k}^{\left(i\right)}\right\}}_{i=1}^{N_{p}}$ denotes $N_{p}$ particles and weights at step *k*. A bootstrap filter propagates particles through the state-transition prior and then updates their weights before resampling:

(8) $$\omega_{k}^{\left(i\right)}\propto p\left(z_{k}|r_{k}^{\left(i\right)}\right)\;\omega_{k-1}^{\left(i\right)},\;\sum_{i=1}^{N_{p}}\omega_{k}^{\left(i\right)}=1.$$

Normalize the weights and resample particles when needed to control weight degeneracy; resampled particles receive equal weights. Proposals other than the transition prior require importance-weight correction. Particle filters represent nonlinear and non-Gaussian distributions, with performance depending on the model, particle count, and computation budget. Navigation-state filtering with RSS observations [24] differs from scalar RSS smoothing. Model and noise choices also affect recursive BLE estimators [25,26]. Position and sensor-fusion applications follow later.

### 4.5. Channel-Aware Filtering

Primary advertising channels 37, 38, and 39 permit separate processing when identifiers are available [12]. Given representative values ${\bar{r}}_{c}$, such as per-channel means over the same observation window, an equal-channel summary is

(9) $${\bar{r}}_{\mathrm{bal}}=\frac{{\bar{r}}_{37}+{\bar{r}}_{38}+{\bar{r}}_{39}}{3}.$$

Pooling weights channels by observed packet count and describes the observed packet mixture; equal weighting gives a channel-balanced summary. Neither is universally unbiased or lower variance, so channel-specific means and missing or uneven sampling require explicit treatment. Filtered channel summaries can be fused; high-percentile or maximum selection remains a heuristic sensitive to extremes. If calibrated aggregates ${\hat{r}}_{c}$ estimate a common RSS level without bias and have independent errors with variances $v_{c}>0$, variance-weighted fusion is

(10) $$\hat{r}=\sum_{c\in C}w_{c}{\hat{r}}_{c},\;w_{c}=\frac{v_{c}^{-1}}{\sum_{\ell \in C}v_{\ell }^{-1}},$$

where C contains the available channels. For $N_{c}$ independent equal-variance samples on channel *c*, the sample-mean variance is $v_{c}=\sigma_{c}^{2}/N_{c}$, with packet-noise variance $\sigma_{c}^{2}$. Temporal or cross-channel correlations require the aggregate-error covariance; raw packet variance alone does not quantify aggregate uncertainty. When channel means contain useful frequency-dependent information, separate features may be preferable to a common-level interpretation.

When channel identifiers are unavailable, we recommend assessing how the aggregation window affects sensitivity to changes in the received channel mixture. Extended Advertising permits auxiliary packets on channels 0–36. Secondary-channel selection for AUX_ADV_IND and AUX_CHAIN_IND is implementation-specific; uniform sampling of all secondary channels is not guaranteed [12] (see the Bluetooth Core Specification, Volume 6, Part B, Section 4.4.2.1). Preprocessing should account for observed channels, unequal sample counts, missing observations, PHY, and transmit-power settings, retaining channel identifiers when the acquisition interface exposes them. Extended-advertising localization has been investigated [27]; further work should assess preprocessing generalization across receivers and acquisition configurations.

Multi-stage preprocessing can combine outlier rejection, averaging, and Kalman filtering [18]; separate fusion approaches integrate Pedestrian Dead Reckoning (PDR) and iBeacon measurements [28]. RSS instability nevertheless remains an open problem and major accuracy limitation [29]. Table 2 classifies studies by preprocessing strategy.

## 5. BLE RSS-Based Positioning

RSS-based positioning comprises three categories: **(i) proximity**, which uses signal presence or the strongest transmitter; **(ii) model-based methods**, which convert RSS to distance using a propagation model; and **(iii) fingerprinting**, which matches observed RSS patterns to a surveyed database. This taxonomy also applies to Wi-Fi, although BLE-specific characteristics produce different performance trade-offs.

Inconsistent error reporting complicates comparisons: Studies report classification percentages, coordinate errors summarized by Mean Squared Error (MSE), mean absolute error (MAE), or other metrics, and less frequently full error distributions or cumulative distribution functions. We identify each approach and its key characteristics, reporting representative results where sufficient information permits comparison. The categories are discussed separately, although practical systems often combine them with other technologies.

### 5.1. Proximity-Based BLE Positioning

Proximity-based BLE localization uses lightweight rules based on signal presence, relative RSS, or thresholds. Low complexity, minimal calibration, and easy deployment come at the cost of coarse resolution and sensitivity to beacon placement, coverage overlap, and user and device effects [73,133]. With asynchronous BLE reception, scan duty-cycling and short-window aggregation help balance responsiveness and stability [120,134].

#### 5.1.1. Presence Detection and Strongest-Beacon Assignment

Presence detection interprets advertisement reception as entry into a predefined area, supporting events, context changes, or handover rather than continuous tracking. Examples include indoor, semi-outdoor, and outdoor context switching [135], mapping beacon identifiers to indoor areas [136], entry/exit notifications [137], and zone-based interaction gating [138]. Temporal aggregation reduces spurious transitions in binary monitoring, such as choke-point entry/exit, without calibration surveys [139].

Strongest-beacon assignment maps users to the region of the transmitter with the highest RSS [120]. Although generally less accurate than lateration or fingerprinting, it suits low-power, low-maintenance deployments [52,140]. Studies use it for room or zone labels [136,141,142,143,144], often prioritizing symbolic locations and waypoint navigation over coordinates [145,146].

Robust variants combine max-RSS, absolute thresholds (e.g., −90 dBm), and windowed majority voting. Windowed strongest-beacon selection has achieved acceptable zone accuracy in small testbeds [147] and high classification rates in long-running clinical and operational deployments [75]. More structured zone recognition can improve room decisions without radio maps [40].

#### 5.1.2. Thresholding and Distance-Proxy Refinement

Threshold rules stabilize proximity decisions by converting fluctuating RSS into categories [148]. They reject weak signals, form RSS bands for room-presence detection [141], or define empirical inside/outside boundaries [149]. Applications include hazard-boundary alerts [150,151,152] and contact detection, where windowed RSS identifies distance violations subject to minimum-duration constraints [153,154]. Supporting reports and poster abstracts are identified separately below.

Path-loss models can supply an RSS-based distance proxy for nearest-beacon or landmark selection while retaining discrete room/zone labels. One-beacon-per-room systems use these models for low-latency detection with minimal calibration [134,142,155]. Others trigger short-range actions using conservative thresholds to limit false positives under motion and obstacles [156,157]. Hybrid methods may apply cell of origin above a high RSS threshold and switch to more complex methods elsewhere to bound worst-case errors [35]. At ultra-short ranges, separable RSS bands enable near-field zone triggering [158].

#### 5.1.3. Integration into Hybrid Pipelines and Practical Limitations

Tracking pipelines can treat stronger RSS as evidence of proximity to an anchor, for example, in particle filters [20,159]. Proximity can initialize or re-synchronize inertial tracking by anchoring a position or defining a bounding box from multiple above-threshold beacons [160]. Landmark detections provide absolute constraints that limit drift and reduce database-generation overhead [161]. Fingerprinting-like schemes can further improve room-level robustness [47].

Hybrid systems switch between confident proximity and fingerprinting [35], preselect candidate regions before refinement [162], or use building geometry to limit leakage across rooms and walls [163]. Dedicated proximity landmarks can enforce doorway or zone transitions when BLE RSS thresholds are ambiguous [164].

Environment and deployment remain decisive [165]. Overlap, interference, and multipath obscure closest-beacon evidence [133], while device orientation and body obstruction affect positioning and contact detection [73,166]. Beacon height, spacing, and coverage-boundary design improve separability and handover reliability [167,168,169,170]. Transmit power and scan/aggregation windows are jointly tuned for latency and robustness: higher power may improve stability while increasing overlap and ambiguity [169,171,172].

#### 5.1.4. Contact Tracing and COVID-19

During COVID-19, digital contact tracing aimed to complement manual tracing by logging short-range encounters and accelerating exposure notifications. Smartphone systems exchange BLE advertisements and assess proximity and duration; their core task is coarse proximity classification [153].

Device diversity, body blockage, and multipath make RSS a noisy distance proxy, causing false positives and negatives. Contact-tracing measurements confirm substantial proximity-estimation variability [173], motivating temporal aggregation and conservative exposure decisions [174].

Privacy and trust also matter at scale. ConTra Corona combines elements of centralized and decentralized cryptographic designs to strengthen privacy [175]. Infrastructure- or wearable-based alternatives (e.g., RFID or Wi-Fi) can benefit controlled environments, with different cost, coverage, and privacy trade-offs from BLE-only smartphones [176].

#### 5.1.5. Proximity: Classification and Reported Performance

Table 3 and Table 4 classify proximity studies and summarize representative results.

### 5.2. Model-Based Positioning

Model-based positioning converts RSS to transmitter–receiver ranges using a propagation model and then combines ranges to estimate location. We review range models, position solvers, and robustness and integration issues, focusing on BLE studies and adaptations of methods shared with other wireless technologies.

#### 5.2.1. RSS-to-Distance Models

BLE Received Signal Strength Indicator (RSSI)-to-distance conversion commonly uses the Log-Distance Path Loss (LDPL) (log-normal shadowing) model. Received power $P_{r}\left(d\right)$ in dBm at distance *d* is modeled as

(11) $$P_{r}\left(d\right)\;=\;A\;-\;10\;n\;\log_{10}\;\left(d/d_{0}\right)\;+\;\xi ,$$

where *A* is the mean received power in dBm at the reference distance $d_{0}$ (typically 1m), n>0 is the dimensionless path-loss exponent, and ξ is a random term in dB. The corresponding range estimate is obtained by inversion:

(12) $$\hat{d}\;=\;d_{0}\;10^{\frac{A-P_{r}\left(d\right)}{10\;n}}.$$

In practice, *A* and *n* are calibrated for each beacon, receiver, or environment; decentralized automatic calibration has also been investigated [219]. As a generic ordinary least-squares example, let $x_{i}=\log_{10}\left(d_{i}/d_{0}\right)$ and $y_{i}=P_{r}\left(d_{i}\right)$ for calibration sample *i*, and let $\bar{x}$ and $\bar{y}$ be their sample means. Provided that the calibration distances are not all identical, Least Squares (LS) yields

(13) $$\hat{n}\;=\;-\;\frac{\sum_{i}\left(x_{i}-\bar{x}\right)\left(y_{i}-\bar{y}\right)}{10\;\sum_{i}{\left(x_{i}-\bar{x}\right)}^{2}},\;\hat{A}\;=\;\bar{y}\;+\;10\;\hat{n}\;\bar{x}.$$

Reported *n* values typically range from 1.2 to 3.0, depending on LoS/Non-Line-of-Sight (NLoS) conditions and the environment [41,44,45,141,220,221,222]. The LDPL model remains the dominant baseline for BLE trilateration and ranging [223].

The log-normal model assumes Gaussian measurement noise in dB:

(14) $$\xi ∼N(0,\sigma^{2}),$$

which gives log-normal received power in linear units. Even with this exact model, inversion is mean-biased: For a=ln(10)/(10n) and known *A* and *n*, $\hat{d}=d\exp\left(-a\xi \right)$ and $E\left[\hat{d}\right]=d\exp\left(a^{2}\sigma^{2}/2\right)$. Thus, the range estimate is median-unbiased but mean-biased. Although convenient for analysis and probabilistic filters, the Gaussian assumption requires validation. Multipath, human-body shadowing, and channel diversity can produce skewed, heavy-tailed, or multimodal BLE RSS distributions [198], adding model-mismatch errors to inversion bias [117,224].

Extensions of standard path-loss models include direct polynomial mappings from RSS to distance, for example,

(15) $$\hat{d}=a+b\;\mathrm{RSS}+c\;{\mathrm{RSS}}^{2},$$

where *a*, *b*, and *c* are fitted coefficients, and RSS is expressed in dBm. Alternatively, one can fit RSS as a function of distance and invert the fitted relationship over a calibrated monotonic interval [52,225,226]. The two regressions minimize residuals in different variables and are not generally equivalent. Piecewise or segmented models capture different distance regimes:

(16) $$\begin{array}{ccc} P_{r}\left(d\right) & =A_{0}-10\;n_{0}\log_{10}\left(d/d_{0}\right)+\xi , & d_{0}\leq d\leq d_{1},\\ P_{r}\left(d\right) & =A_{1}-10\;n_{1}\log_{10}\left(d/d_{1}\right)+\xi , & d>d_{1}, \end{array}$$

Here, $d_{0}>0$ is the first reference distance and $d_{1}>d_{0}$ is the transition distance. The dimensionless parameters $n_{0}$ and $n_{1}$ are the path-loss exponents in the first and second regimes. The intercepts $A_{0}$ and $A_{1}$ are the reference mean received powers in dBm for the first regime at $d_{0}$ and for the second regime extrapolated to $d_{1}$, respectively. For a continuous mean path-loss curve, the intercepts must satisfy $A_{1}=A_{0}-10n_{0}\log_{10}\left(d_{1}/d_{0}\right)$. Fitting the intercepts independently allows a discontinuity at $d_{1}$, which must be justified by the modeled propagation transition rather than assumed implicitly.

Structural extensions add deterministic losses:

(17) $$P_{r}\left(d\right)\;=\;A-10\;n\log_{10}\left(d/d_{0}\right)\;-\;L_{\mathrm{walls}}\;-\;L_{\mathrm{floor}}\;-\;L_{\mathrm{body}}\;+\;\xi ,$$

where $L_{\mathrm{walls}}=\sum_{j}\kappa_{j}N_{j}$ sums penetration losses: *j* indexes wall/material classes, $N_{j}$ is the number of traversed walls in class *j*, and $\kappa_{j}$ is the corresponding loss in dB. The terms $L_{\mathrm{floor}}$ and $L_{\mathrm{body}}$ are floor and human-occlusion losses in dB [51,108,132,227,228,229]. An empirical oscillatory correction can represent local departures from a smooth path-loss curve, for example,

(18) $$P_{r}\left(d\right)=A-10n\log_{10}\left(d/d_{0}\right)+A_{s}\cos\;\left(\frac{2\pi d}{\ell_{s}}+\phi \right)+\xi ,$$

where $A_{s}$ is the fitted oscillation amplitude in dB, $\ell_{s}>0$ is a fitted spatial period in the same length unit as *d*, and ϕ is a phase offset in radians. This empirical expression is not a physical two-ray derivation: physical interference depends on the difference between propagation-path lengths and on the summed complex fields. Two-ray and ray-tracing approaches explicitly account for reflection geometry [31,230,231]; their performance and transferability depend on the deployment.

Channel-aware approaches exploit BLE advertising channels [118]. Separate models for m∈{37,38,39} give

(19) $$P_{r}^{\left(m\right)}\left(d\right)\;=\;A_{m}-10\;n_{m}\log_{10}\left(d/d_{0}\right)+\xi_{m},\;{\hat{d}}_{m}\;=\;d_{0}\;10^{\frac{A_{m}-P_{r}^{\left(m\right)}\left(d\right)}{10\;n_{m}}},$$

For independent, unbiased range estimates with error variances $v_{m}>0$, inverse-variance fusion gives

(20) $$\hat{d}=\frac{\sum_{m}w_{m}{\hat{d}}_{m}}{\sum_{m}w_{m}},\;w_{m}\propto v_{m}^{-1},\;v_{m}=\mathrm{Var}\left({\hat{d}}_{m}-d\right).$$

These are range-error variances, not RSS-noise variances. For small RSS errors and fixed calibration parameters, first-order propagation gives

(21) $$v_{m}\approx {\left[\frac{\ln(10)}{10n_{m}}{\hat{d}}_{m}\right]}^{2}\sigma_{m}^{2},$$

where $\sigma_{m}^{2}=\mathrm{Var}\left(\xi_{m}\right)$ refers to the RSS value or aggregate used in that range estimate. This approximation omits calibration uncertainty and nonlinear effects. Biased or correlated estimates require corresponding bias and covariance treatment; the naive LDPL inversion does not automatically satisfy the unbiasedness assumption. Separately, the distribution of packet RSS pooled across channels obeys the law of total variance. If *R* denotes a packet-level RSS value and *M* its channel index,

(22) $$\mathrm{Var}\left(R\right)\;=\;E\;\left[\mathrm{Var}\;\left(R\;|\;M\right)\right]\;+\;\mathrm{Var}\;\left(E\;\left[R\;|\;M\right]\right),$$

The second term is the between-channel contribution to packet-mixture variance. An averaged estimator’s variance also depends on sample counts and temporal or cross-channel correlations. Studies report lower variance and more consistent path-loss parameters with separate channels than aggregated RSS [109,118,231,232,233]. Limitations of a single Gaussian log-normal model motivate hybrid path-loss, channel-modeling, sensor-fusion, and probabilistic-learning methods [234,235,236,237,238]. Stochastic BLE RSSI modeling also addresses time-correlated noise jointly with path-loss parameter estimation [239]. The BLE Physical Layer (PHY) configuration affects ranging: a study of coded PHY with S=8 evaluates RSSI-based distance estimation alongside RTT and jitter [240].

Table 5 distinguishes RSS-to-distance mappings from RSS-field prediction and joint localization/mapping. A model that predicts RSS at a position is not automatically a direct range estimator. Supporting reviews and poster abstracts are not classified as primary model contributions.

#### 5.2.2. Position Solvers

Range or distance-like estimates are combined to locate an unknown node, as in GNSS and Wi-Fi positioning [282]. We summarize lateration fundamentals and reported BLE adaptations.

Lateration estimates the position $p\in R^{d}$ from distances between a receiver and multiple anchors. Spherical lateration uses absolute range estimates, whereas hyperbolic lateration uses metric range differences between anchor pairs. RSS-difference models instead yield distance ratios under the assumptions stated below.

##### Range-Based Lateration (Spherical)

Given *m* anchors at known positions $b_{i}$, i=1,…,m and noisy range estimates ${\hat{d}}_{i}$ (often derived from RSS or other BLE measurements), a standard formulation is weighted LS:

(23) $$\hat{p}=\arg\min_{p}\;\sum_{i=1}^{m}w_{i}\;{\left(∥p-b_{i}∥-{\hat{d}}_{i}\right)}^{2},$$

where $w_{i}$ reflects link reliability or geometric relevance. In 2D, with x=(x,y) and anchors $x_{i}=\left(x_{i},y_{i}\right)$, the geometric range is $d_{i}=∥x-x_{i}∥$.

This RSS → distance → position pipeline includes trilateration or circle intersections [87,110,147,247,254], multilateration with more than three anchors [44,77,220,251,261], and integration into tracking pipelines [90,283].

With three beacons, range estimates ${\hat{d}}_{1},{\hat{d}}_{2},$ and ${\hat{d}}_{3}$ define three circles:

(24) $${∥x-x_{k}∥}^{2}={\hat{d}}_{k}^{2},\;k\in \left\{1,2,3\right\}.$$

Their common intersection gives the position estimate when the ranges are consistent; otherwise, a best-fit solution is required. BLE implementations use circle intersections or equivalent closed-form algebra after converting RSS to ranges [32,87,96,147,247,254].

With *n* beacons, robust aggregation of multiple trilateration candidates requires enough uncontaminated, well-conditioned triplets; ill-conditioned triplets should be excluded or down-weighted. Ordinary averaging alone does not ensure robustness. Weighting based on the sample median and mean absolute deviation about that median combines all n3 candidates in [55]; similar multi-candidate aggregation appears in [284].

Subtracting one squared anchor-range equation from the others produces a linear system Ax≈b, where *A* contains known anchor-coordinate differences and b contains the corresponding squared-range and squared-anchor-coordinate terms. With *m* anchors in *d* spatial dimensions, this reference-subtracted system has m−1 equations and is overdetermined when m−1>d: at least four anchors in 2D or five in 3D. Redundancy can improve robustness, although additional biased ranges do not guarantee an improvement [77,236,241]. If *A* has full column rank and the weight matrix *W* is positive definite, weighted LS gives

(25) $$\hat{x}={\left(A^{⊤}WA\right)}^{-1}A^{⊤}Wb.$$

Diagonal weights $W=\mathrm{diag}(w_{i})$ can encode chosen link reliabilities. Subtracting a common anchor equation induces correlated residuals, however; generalized least squares instead uses *W* proportional to the inverse residual covariance when that covariance is known [282]. In practice, weighted fits commonly emphasize short or reliable links [52,89,241,271,285].

Performance depends strongly on calibration and geometry [286]. An RSS-to-range least-squares pipeline with EKF/PDR fusion and graph-estimated beacon positions reports mean errors of 3.25 m (dense) and 4.69 m (sparse) [17]; other studies examine calibrated lateration and geometry-dependent corrections [77,89]. In [236], average errors of about 1.1 m with 11 beacons and 1.6 m with six illustrate density and geometry effects.

Instead of linearization, lateration can be solved directly as a Nonlinear Least Squares (NLLS) problem:

(26) $$S\left(x\right)=\sum_{i=1}^{m}{\left(∥x-x_{i}∥-{\hat{d}}_{i}\right)}^{2},\;\hat{x}=\arg\min_{x}S\left(x\right).$$

Here, x is the candidate position, $x_{i}$ is the known position of anchor *i*, ${\hat{d}}_{i}$ is its RSS-derived range estimate, and *m* is the number of anchors. Iterative NLLS variants are evaluated in [25,261].

Weights $w_{i}$ encode both link reliability (stability, outlier filtering, and channel effects) and geometric considerations (visibility and spatial diversity). This unified view covers LS/WLS, NLLS, closed-form trilateration, and region-based methods when exact intersections are unreliable [123,241].

Weighting strategies include (i) inverse-distance or expected-error weights to limit far-link error amplification [241,285,287]; (ii) reliability weighting and device selection through decision rules, outlier rejection, or channel-dependent weights [271]; (iii) temporal attenuation favoring recent measurements [288]; and (iv) robust reweighting, such as likelihood/M-estimator-like schemes, to suppress anomalies [289,290]. Weighted-centroid approaches similarly improve geometric consistency without trusting every RSS-derived range equally [36,135,291].

##### RSS-Difference and Range-Difference Formulations

Assume that the links share a path-loss exponent η and that their reference-power intercepts are equal or have been calibrated to a common value. Under the log-distance model, the difference between the resulting RSS measurements $r_{i}$ and $r_{j}$, expressed in dBm, is

(27) $$\Delta r_{ij}=r_{i}-r_{j}=-10\eta \log_{10}\;\left(\frac{d_{i}}{d_{j}}\right)+\nu_{ij},\;d_{i}=∥x-s_{i}∥,$$

where x is the receiver position, $s_{i}$ is anchor *i*’s position, and $\nu_{ij}$ is the difference between the two RSS errors. Independent transmit-power or hardware offsets do not cancel unless accounted for by calibration. Neglecting noise gives the distance ratio

(28) $$q_{ij}=10^{-\Delta r_{ij}/\left(10\eta \right)},\;∥x-s_{i}∥=q_{ij}∥x-s_{j}∥.$$

For distinct anchors, a fixed ratio defines an Apollonius circle in 2D or a sphere in 3D; when $q_{ij}=1$, it defines the perpendicular bisector or bisector plane. A compatible estimator fits the RSS differences directly:

(29) $$\hat{x}=\arg\min_{x}\sum_{(i,j)\in P}w_{ij}{\left[\Delta r_{ij}+10\eta \log_{10}\frac{∥x-s_{i}∥}{∥x-s_{j}∥}\right]}^{2},$$

where P is the chosen set of anchor pairs, and $w_{ij}>0$ are reliability weights. Pair differences sharing an RSS measurement have correlated errors, so statistically efficient weighting should account for their covariance. Temporal aggregation may precede differencing but does not change the ratio geometry.

##### Range-Difference Lateration (Hyperbolic)

Hyperbolic constraints require metric range differences, $\Delta d_{ij}=d_{i}-d_{j}$, not directly supplied by RSS ratios. The algorithm termed “hyperbolic” in [282] instead subtracts squared, individually estimated ranges to form a linear system. Collaborative smartphone approaches also combine RSS-derived lateration with fingerprinting in hybrid, cooperative positioning [292].

#### 5.2.3. Robustness Issues and Integration

For biased ranges, annuli can replace circles to define a feasible region [119]. Min–max or bounded-box solvers use the *k* best beacons and shrink this region as scans arrive, improving stability under fragile RSS-to-range inversion [123]. Nikodem combines RSS-derived annular constraints with measurements aggregated over non-overlapping 30 s windows and total-signal-strength weighting [119]; other approaches refine feasible regions through evidence accumulation [79,80].

Noise or bias can prevent circle intersections. Remedies include proportional radius correction and projection onto trusted constraints, such as short or LoS links [89]. Perpendicular-bisector constructions can stabilize initialization; explicit non-intersection rules appear in [113], and LS pipelines also use proportional radius adjustment [293].

Anchor geometry determines how range errors affect position: angularly diverse, non-collinear placement improves conditioning, whereas corridors may promote collinearity. Selection should balance geometry and link quality [84,294]. The top-*N* closest or most reliable beacons can outperform all visible anchors [49], because weak links may add bias without sufficient geometric benefit [287]. Four rather than three anchors can reduce degradation near polygon edges and smooth transitions [36,295].

Lateration provides direct position fixes or measurement models for recursive estimators (PF, KF/EKF) that constrain drift and stabilize trajectories [95,248,296]. To improve RSS-to-distance mapping, studies use per-beacon calibration [89,297], transmit-power-aware ranging [275], channel-aware ranging [271], and two-ray [270] or segmented propagation models [236].

#### 5.2.4. Model-Based Positioning: Classification and Reported Performance

Table 6 and Table 7 classify model-based studies and summarize selected results.

### 5.3. Fingerprinting

Following [312], fingerprinting comprises (i) deterministic *k*-Nearest Neighbors (*k*-NN)/Weighted *k*-Nearest Neighbors (W*k*-NN) methods; (ii) probabilistic methods modeling measurements conditional on position; and (iii) machine learning methods. Probabilistic approaches are less extensively explored than in Wi-Fi [313]. Channel-aware features apply across all three families and form an orthogonal attribute in Table 8.

#### 5.3.1. Lazy Learning Methods

Classic *k*-NN/W*k*-NN remains a strong baseline due to simplicity and robustness, often with Euclidean (or Manhattan) distances and inverse-distance weights [13,42,314,315,316,317,320,342,402]. Formally, given an online RSS vector $r\in R^{B}$ with *B* RSS features and a reference fingerprint $f_{i}$ at location $x_{i}$, typical distances are

$$d_{i}^{\left(\ell_{2}\right)}=∥r-f_{i}{∥}_{2},\;d_{i}^{\left(\ell_{1}\right)}={∥r-f_{i}∥}_{1},$$

Let $d_{i}$ denote the selected fingerprint distance, and let $N_{k}$ denote the indices of the *k* nearest reference fingerprints. Then, *k*-NN/W*k*-NN uses inverse-distance (or power-law) weights

$$w_{i}=\frac{1}{{\left(d_{i}+\varepsilon \right)}^{p}},\;p\geq 0,\;\varepsilon >0,\;\hat{x}=\frac{\sum_{i\in N_{k}}w_{i}\;x_{i}}{\sum_{i\in N_{k}}w_{i}}.$$

Setting p=0 yields unweighted *k*-NN, whereas ε>0 prevents division by zero.

Recent BLE RSS W*k*-NN methods constrain candidates using previous positions and adaptive thresholds to reduce error propagation and search complexity [403]. Distance metric, *k*, and weighting should be tuned jointly for each dataset before comparing optimized methods [404]. Transmit power, beacon density, and measurement windows also affect accuracy [171,244,314,315,395].

The density study in [14] reports 95th-percentile errors below 2.6 m for high-density BLE and 4.8 m for lower density. A generic alternative to inverse-distance matching uses Gaussian similarity weights. For $d_{i}={∥r-f_{i}∥}_{2}$,

(30) $$u_{i}=\exp\;\left(-\frac{d_{i}^{2}}{2\sigma^{2}}\right),\;\hat{x}=\frac{\sum_{i}u_{i}\;x_{i}}{\sum_{i}u_{i}},$$

where $u_{i}$ is an unnormalized similarity weight, and σ>0 is a kernel scale in the same units as $d_{i}$. Normalizing these weights does not by itself establish a probabilistic likelihood or posterior; a Bayesian interpretation additionally requires an observation model and prior. Operational parameters, including advertising period, observation window, fingerprint dimensionality, beacon density, and transmit power, should be reported alongside positioning results.

##### Spatial Template Matching

Eight-neighborhood template matching (ENTM) builds access-point-specific 3×3 templates from measured RSS and learned quadrant-specific center-to-neighbor differences. It selects the position that minimizes the squared differences between these templates and radio-map subimages [352]. The reported RMSE was 1.0 m versus 1.2 m for both *k*-NN and W*k*-NN [352].

#### 5.3.2. Machine Learning in Fingerprinting

Classical methods classify rooms or grid cells or regress coordinates using support-vector, random-forest, linear-discriminant, and decision-tree models [53,316,353,405]. Linear support vector machines (SVMs), for example, maximize class separation while regularization permits some misclassifications. Calibrated classifier probabilities can support fusion if training priors and dependence are accounted for; performance depends on the task, data, and scenario. XGBoost has also been evaluated [371]. Room-level classification serves assistive living and building automation, where semantic labels suffice [355,406,407].

Semi-supervised physics-informed genetic fuzzy systems have been compared with neural-network and *k*-NN baselines for BLE localization [374].

Deep-learning approaches include denoising autoencoders for reconstructing clean RSS patterns from corrupted inputs [383]. Xiao et al. use reconstruction-based denoising-autoencoder scores for weighted fingerprint matching, reporting approximately 1.09 m mean horizontal localization error in a 9.6 m × 17.5 m room with ten beacons [375]. Semi-supervised deep reinforcement learning (DRL) with variational autoencoders (VAEs) uses unlabeled fingerprints to reduce reliance on fully labeled maps, reporting a 23% improvement in distance to the target over supervised DRL [380]. Synthetic fingerprints and generative adversarial network (GAN) augmentation aim to reduce surveys and improve generalization [376,389]. Dynamic-environment pipelines combine interpolation, differential RSS features, Transformer representations, and outlier mitigation [408]. Machine-learning algorithms have also been evaluated for BLE RSSI-based indoor localization [409].

#### 5.3.3. Channel Diversity in Fingerprinting

Separating BLE advertising channels into radio maps or “virtual beacons” can improve fingerprinting over channel aggregation [52,348,350,351]. A simple formulation builds three models ${\left\{M^{\left(m\right)}\right\}}_{m\in \{37,38,39\}}$ and fuses their position estimates. If their errors are unbiased, independent across channels, and isotropic with covariance $C_{m}=v_{m}I$ ($v_{m}>0$), then

$$\hat{x}=\frac{\sum_{m}\alpha_{m}{\hat{x}}^{\left(m\right)}}{\sum_{m}\alpha_{m}},\;\alpha_{m}=v_{m}^{-1},$$

where *I* is the identity matrix, and $v_{m}$ is the per-coordinate position-error variance. Anisotropic or correlated position errors require covariance-aware fusion, and overlapping observations should not be treated as independent without justification. Alternatively, per-channel RSS can be concatenated as $r=[r^{\left(37\right)};r^{\left(38\right)};r^{\left(39\right)}]$ and used in standard *k*-NN/W*k*-NN matching [34]. For example, [350] reports a 28.5% reduction in 90th-percentile positioning error, from 2.81 m to 2.01 m, using separate-channel fingerprints.

#### 5.3.4. Limitations and Integration

Radio maps require maintenance as environments and measurement conditions change. Online updates, including dynamic resampling and cut-off areas, remove unreliable RSS observations [390,410]. Radial-basis functions, kernel regression, and Gaussian-process-like methods densify sparse surveys or assign probabilistic location scores [14,37,162,336]. Fuzzy clustering and weighted interpolation also aim to improve BLE radio maps [411].

Performance depends on RP density, beacon count, user orientation, and device heterogeneity [33,140,314,315,412]. Orientation models, per-orientation fingerprints, and iterative candidate restriction aim to reduce error and computation [33,35,57,412,413,414]. For room-level BLE RSSI localization, gap-aware segmentation with random-forest and hidden Markov model (HMM) inference handles irregular RSSI streams and discontinuous movement [415].

#### 5.3.5. Classification and Reported Performance

Table 8 classifies the fingerprinting studies above; Table 9 summarizes selected reported results.

## 6. Sensor Fusion Approaches

Fusion combines BLE-based positioning with other technologies to improve accuracy or provide continuous position estimates. A recurring combination in the reviewed literature pairs any of the three BLE approaches with pedestrian dead reckoning (PDR). Other schemes integrate Wi-Fi, GNSS, or both, sometimes adding environmental or map information to refine estimates.

### 6.1. Integration with PDR

PDR integrates step events and heading changes from inertial sensors to estimate short-term motion. BLE trilateration, proximity, or fingerprinting provides absolute updates to correct drift and often initialize or re-anchor trajectories [28,74,84,285,296,418]. Fusion typically uses Bayesian filtering—most commonly Kalman/EKF variants or particle filters—with PDR providing predictions and BLE measurement corrections.

Reported outcomes include smoother trajectories and consistent error reductions; Kalman fusion of BLE trilateration and dead reckoning achieved sub-meter accuracy in the evaluated testbed [84].

### 6.2. Other Fusion Approaches

For conditionally independent modalities, Bayesian fusion combines observation likelihoods with a prior over the candidate state. Classifier scores require calibration or an explicit score-fusion model; posterior outputs also require accounting for training priors. Table 10 distinguishes complementary online inputs from offline training/calibration and benchmark ground truth. Optical laser/single-photon avalanche diode (SPAD) proximity sensors add constraints [164], whereas BeSound uses ultrasound supervision during training and BLE-only inference [65]. Comparing separate systems does not itself constitute fusion.

Vision can complement BLE RSS region classification: Mamede et al. combine RSSI and camera-image classifier outputs through ensemble models [419]. Near-Field Communication (NFC) tags can provide known-location checkpoints complementing BLE guidance in systems supporting indoor/outdoor navigation [136].

Contextual and environmental signals further stabilize positioning. Phone orientation sensors can reference RSS-derived bearings to north [72]; orientation and radio bearing are distinct observables, and a magnetometer does not directly measure a BLE signal’s arrival direction. Barometric pressure is widely used for multi-floor inference by mapping changes to relative altitude, enabling reliable floor-transition detection with BLE or inertial tracking [420]. Building/floorplan constraints and, in some studies, learned motion/velocity priors such as ResNet-18 prune implausible trajectories and reduce drift [235]. Smart-home contact and pressure sensors provide location cues that particle filters can fuse with BLE RSS for greater robustness [234].

Fusion architecture and measurement gating can matter as much as the sensor set. BLE proximity events provide anchor-like corrections to reset PDR drift when users are confidently near beacons [28]. Other systems switch between fingerprinting and multilateration according to beacon visibility and signal conditions [285]. Channel-aware designs weight BLE measurements by their estimated reliability (e.g., down-weighting higher-variance channels) before combining position hypotheses. For emerging cellular-based positioning, recent work fuses 5G uplink signals with complementary BLE RSSI through spatiotemporal attention [421].

**Table 10:** BLE multimodal approaches: online fusion, offline calibration/training, and benchmarking.

Category (Sensors)	Fusion Strategy	Role/Interpretation	Refs.
BLE RSS + PDR	KF/EKF/UKF, PF; adaptive covariances; landmark updates	**Online input.** EKF/KF/PF drift correction; adaptive noise/covariance and beacon/zone resets improve stability.	[49,51,118,160,285,294,307,418,422,423,424,425,426,427,428,429,430]
PDR-assisted RSS surveying	Joint anchor and path-loss calibration	**Offline calibration; subsequent online localization.** PDR assists surveying to estimate Wi-Fi/BLE anchor positions and interfloor path-loss parameters.	[431]
BLE RSS + Map Constraints	PF/UKF; particle pruning; wall or floor constraints	**Static prior.** PF/UKF constrained by maps, walls or floors.	[162,417,432,433]
BLE RSS + Wi-Fi RSS	Feature-level fusion; priors; candidate pruning	**Online radio input; offline map if required.** BLE/Wi-Fi zone priors, candidate pruning, and combined fingerprints/databases improve robustness.	[259,376,378,412,434,435,436,437]
GNSS + BLE, with PDR or NFC	Indoor/outdoor handover; tightly coupled navigation	**Online input/checkpoints.** GPS/BLE/NFC indoor/outdoor guidance; GNSS/PDR/BLE pedestrian navigation in dense urban settings.	[136,296]
BLE RSS + UWB ranging	EKF fusion of ranging information	**Online ranging input.** BLE path-loss distances fused with UWB ranges.	[438]
BLE RSS + acoustic ranging	KF fusion or on-demand refinement	**Online acoustic input.** Acoustic ToF/TDoA refines coarse BLE tracking on demand to centimeter-level accuracy via switching or fusion.	[50,439]
BLE RSS + vision or markers	Study-specific gating, regression or calibration	**Study-dependent role.** Online visual input and offline RSS-map construction/calibration are distinct uses; only the former is online fusion.	[134,199,320,440,441,442,443]
BLE RSS + ambient or context sensors	PF/confidence fusion; plausibility checks	**Online contextual input.** Confidence fusion, plausibility checks, and motion-aware weighting reduce false room switches; power/sound feature fusion improves presence inference.	[76,114,161,162,234,325,356,359,416,420,444,445]
BLE RSS + AoA	Kalman-based fusion or learned joint RSS/AoA inference	**Online radio input.** Study-specific Kalman fusion, learned joint features, or other estimators; Kalman filtering is not universal.	[293,446,447]
BLE RSS + optical proximity	Optical laser/SPAD time-of-flight constraints	**Online proximity input.** Optical detections constrain BLE estimates; optical ToF differs from UWB ranging.	[164]
BLE with acoustic supervision (BeSound)	Cross-modal knowledge distillation	**Offline training supervision.** Ultrasound supervises BLE training; BLE-only inference excludes online acoustic/Kalman fusion.	[65]
RSS and AoA dataset	Joint recording for algorithm evaluation	**Benchmark/ground truth.** Joint observables do not establish a Kalman or other fusion algorithm.	[448]

## 7. BLE RSS Datasets

Environment-dependent performance complicates comparisons of BLE RSS-based positioning methods. Well-documented open datasets support reproducibility and fair comparison through shared data, protocols, and metrics. Following documentation and organization practices established by Wi-Fi datasets such as UJIIndoorLoc [449], BLE datasets address diverse objectives. Early BLE RSS datasets mainly targeted university fingerprinting: Ref. [450] examined device heterogeneity and transmit power, while Ref. [394] compared power settings and receiver- versus transmitter-localization schemes.

Office and academic datasets cover dense grids, multiple power levels, autonomous or remote modes, smartphone heterogeneity, energy-aware acquisition, and LiDAR-calibrated maps. The ISTI-CNR dataset [394] includes a research-office grid with multiple power settings and acquisition modes for fingerprinting and tracking. SAL-RB [451] records BLE RSSI from ten nRF52840 sniffers at 100 ms intervals in an automotive testbed, with walking/vehicle trajectories and photosensor-derived region labels. A LiDAR-equipped robot supplies geometric ground truth for dense apartment/building radio-map calibration [279]. Clinical BLE tag measurements from fixed Raspberry Pi receivers provide labeled RSSI images for deep-learning localization [452].

Channel-aware datasets include RSS and channel measurements for BLE 5.0 Extended Advertising [27]. Auxiliary advertisements can use secondary channels 0–36 alongside primary advertising on channels 37–39; these remain distinct channel classes, and a measurement window need not sample every channel [12]. Studies [351,453] compare systems with and without channel information using custom hardware, including frequency-dependent directive antennas. Within the reviewed corpus, we found no openly accessible smartphone dataset pairing per-packet advertising-channel identifiers with RSS. OutFin combines outdoor Bluetooth, Wi-Fi, cellular, and inertial fingerprints with location ground truth; its inquiry-based discovery and device-capability flags do not establish a BLE-advertising subset, so it remains a contextual multimodal benchmark [454]. Indoor multi-technology *corpora* support cross-technology comparisons [247], while a recent industrial, interference-prone dataset combines BLE/Wi-Fi RSSI with position, orientation, and velocity ground truth [455].

Outside the positioning context, some datasets have focused on RSS analysis to study specific effects. LOS/NLOS and proximity studies include controlled experiments (indoor/outdoor) with obstacles and per-channel logging [117,166], as well as labeled Wi-Fi and BLE RSS measurements for training and evaluating NLOS detectors [456,457].

Table 11 separates study/descriptor citations from repository links; a dash denotes that no separate paper is cited. Counts refer to different units, including raw observations, fingerprints, images, positions, transmitters, and advertising slots. Competition entries identify specific tracks or releases. IPIN 2019 Track 3 and OutFin are retained as contextual multimodal benchmarks rather than BLE RSS-only datasets. The final column separates auxiliary measurements from analysis methods.

## 8. Application Domains of BLE-Based Positioning

BLE positioning spans geofencing, visitor services, healthcare, safety-critical monitoring, and industrial workflows, supported by low deployment cost, off-the-shelf smartphone compatibility, and adaptability to diverse environments [496]. This section groups applications by domain, independently of the positioning technique. Proximity methods suit simple zone or encounter detection with low computational requirements.

Applications of BLE navigation and user assistance [256,497] include airport luggage tracking and passenger notifications [137]. RSSI has also been investigated for detecting close contacts between ship passengers [498]. Related services include parking guidance and vehicle-recall functions that help users locate their cars [200,499].

Healthcare and assisted living [191,500,501] include patient monitoring in hospitals and nursing homes [38,40,62], sedentary behavior analysis [241,502], and home rehabilitation and elderly care [40]. Assistive navigation for visually impaired users often uses BLE in hybrid trilateration or fingerprinting solutions [503,504]. During COVID-19, BLE proximity became central to contact tracing and exposure notifications; some studies used channel-specific responses to improve distance estimates [117,231].

Safety and emergency applications include fire management, evacuation support and command-center firefighter tracking [505,506], access and security monitoring [86], and evacuation guidance for elderly-care facilities [507]. Other uses include crowdsensing at large gatherings [508], AGV proximity control in crematorium workflows [199], and infant monitoring with dynamically defined danger zones and entry alerts [297].

In shopping malls and retail environments, BLE is used for proximity-based marketing through broadcast advertisements [357,509]. BLE-based positioning has been integrated into power management systems for large buildings [510,511] and smart homes [512], enabling occupancy-driven control of heating, ventilation, and air conditioning (HVAC), and lighting [39,141]. These applications demonstrate BLE’s ability to improve energy efficiency and support context-aware smart building services.

Industrial and agricultural applications also use BLE positioning. In warehouses, it supports logistics optimization and forklift tracking [402]. In agriculture, BLE tags enable continuous livestock monitoring and estimation of drinking-event frequency and duration [30,513]. BLE has also been applied to study group dynamics, such as the co-localization of walking groups to analyze collective mobility behavior [514].

Construction, mining, and industrial systems use BLE to track workers and equipment, issue proximity warnings, associate assets with operators, and support logistics and digital site monitoring under obstruction and metallic interference [515,516,517,518,519,520,521]. These real-time locating systems (RTLSs) often prioritize robustness and low maintenance over fine-grained accuracy.

Education-related deployments use BLE beacons for automated attendance tracking and location-triggered classroom services, including personalized notifications and interactions between lecturers and students [522,523]. BLE has been deployed in museums and cultural heritage sites to provide context-aware visitor guidance and personalized content delivery [73,192,225,524].

Smartphones and wearables can continuously scan BLE advertisements for coarse proximity indicators, including RSSI trends and encounter events. Systems can aggregate these opportunistic observations across participants to estimate pedestrian density and flow, identify popular routes, and detect congestion hotspots without dense dedicated infrastructure [525,526]. Typical implementations use beacon sightings or encounter logs to reconstruct coarse trajectories and activity patterns in public buildings, campuses, and large events [527,528]. Challenges include device-dependent scanning, uneven participation, and the privacy implications of collecting encounter and trajectory data.

Table 12 summarizes the application domains discussed in this section.

## 9. Comparison with Other Technologies

We compare BLE RSS positioning with RSS-based alternatives, excluding timing- or angle-based methods (e.g., ToF and Angle of Arrival (AoA)). Because many methods originated in Wi-Fi or classic Bluetooth, we emphasize direct comparisons with BLE. The technologies considered include Wi-Fi, LoRa/LoRaWAN, UWB, Zigbee, classic Bluetooth, NFC, and Radio-Frequency Identification (RFID).

Table 13 retains study-specific metrics and conditions; its results are neither universal accuracy ranges nor a ranking across deployments. NFC and passive RFID provide contextual proximity or identification alternatives where coordinate-based RSS comparisons are unavailable.

Wi-Fi, an IEEE 802.11 Wireless Local Area Network (WLAN), typically operates at 2.4 GHz and 5 GHz, with newer versions using 6 GHz (Wi-Fi 6E/7). Indoor RSS depends on multipath, users, devices, and available Access Points (APs). Wi-Fi generally consumes more energy than BLE but can reuse infrastructure. Direct RSS comparisons include [247,611,612,613]. Faragher and Harle reported 95th-percentile errors below 2.6 m for BLE and 8.5 m for Wi-Fi in the same area [14]. De Blasio et al. reported Wi-Fi mean errors of 3.94 m to 4.82 m (90th percentile: 5.21 m to 7.0 m) and BLE mean errors of 1.47 m to 2.15 m (90th percentile: 1.81 m to 3.58 m) with 1 beacon per 45.7 m^2^ [33].

Classic Bluetooth is a 2.4 GHz ISM wireless personal area network (WPAN) technology for continuous connections, including audio and peripherals. RSS/RSSI supports proximity and coarse positioning, but discovery and connection procedures make dense beacon deployments less practical than BLE advertising. Reported results include 2.2 m positioning error with smoothed BLE iBeacon measurements [614] and 5.1 m mean horizontal error for classic Bluetooth RSSI fingerprinting (3.90–5.78 m in static tests) [608].

Zigbee is a low-power stack built on IEEE 802.15.4, typically at 2.4 GHz. Indoor RSS performance depends on anchor density and geometry; smartphone support is less common than for BLE. A multi-radio RSS trilateration study reported BLE/Zigbee overall mean errors of 0.753 m/0.896 m and 0.661 m/0.882 m in Environment 1 and 0.844 m/0.911 m in Environment 2 [247].

LoRa/LoRaWAN provides low-power, long-range, low-bitrate links, usually in sub-GHz ISM bands such as 868 MHz or 915 MHz. Sparse gateways and slow indoor RSS sampling often yield coarser localization than BLE. In [247], the LoRaWAN aggregate of 1.190 m conflicts with the two environment-specific values, 0.846 m and 1.109 m; we neither use that aggregate nor replace it with a calculated value. Separately, wide-area LoRaWAN RSSI fingerprinting reported approximately 284.57 m mean error [615].

Although usually used for accurate ranging, short-range, wide-bandwidth UWB also supports RSS-only proximity and fingerprinting. Its RSS remains sensitive to multipath and device differences, and RSS-only studies are less common than for BLE. A direct BLE/UWB RSS fingerprinting comparison appears in [609], with broader context in [1].

NFC uses near-field induction at 13.56 MHz for identification, access control, and pairing. Its centimeter-scale range provides proximity rather than room-scale RSS positioning. BLE at 2.4 GHz covers meters to tens of meters for zone detection and coarse positioning. Short-range BLE as a near-field-like alternative is studied in [158]; NFC proximity comparisons appear in [192].

RFID includes LF/HF systems (including 13.56 MHz) and UHF systems (typically 860 MHz to 960 MHz) used for inventory and asset tracking. Received-power or link-quality indicators can support presence, zones, and sometimes RSS localization depending on the band and reader/tag design. Often-passive, inexpensive RFID tags generally require dedicated readers; active BLE tags can use commodity receivers. Passive UHF RFID and BLE are compared for RSS-based room detection and contact tracing in [176], with broader context in [1].

Beyond this review’s RSS scope, BLE provides AoA, AoD, and MCPD positioning observables [616,617].

## 10. Open Challenges and Future Trends

BLE RSS supports proximity, fingerprinting, and model-based positioning on accessible hardware, but acquisition, propagation, and evaluation conditions affect performance. Priorities include cross-device/environment robustness, affordable maintenance, and integration with additional Bluetooth observables.

Multipath, body obstruction, and antenna orientation alter RSS without distance changes. Channel diversity and filtering can reduce variability [108], but methods should distinguish transient fluctuations from persistent bias and quantify reliability. Fingerprinting priorities include device normalization, adaptation with limited labels, and incremental radio-map updates, evaluated across independent devices, sessions, and environments. Physics-informed models can combine propagation constraints with learned corrections, but they must demonstrate gains over simpler calibrated baselines under imperfect propagation assumptions.

Extended Advertising provides channel diversity already investigated for localization [27], but observations may be incomplete, unevenly sampled, or inaccessible through receiver interfaces. Datasets should document available channel identifiers, PHY, transmit power, timestamps, and filtering settings. Comparisons should control observation duration, packet count, and energy to distinguish diversity gains from increased acquisition effort.

Bluetooth Core 5.1 introduced Direction Finding in 2019, using receiver antenna arrays for Angle of Arrival (AoA) or transmitter arrays for Angle of Departure (AoD) [616]. Core 6.0 introduced Channel Sounding (CS) in 2024 [618], supporting multi-frequency Phase-Based Ranging (PBR) and Round-Trip Timing (RTT) [619]. Unlike connectionless RSS observation, standardized CS requires compatible controllers at both endpoints and an LE connection for setup and control [619]. Earlier RSS/multi-carrier phase-difference comparisons predate standardized CS [617]. These direction and distance observables remain affected by multipath, non-line-of-sight propagation, antenna characteristics, and calibration; evaluations should distinguish ranging from positioning errors and account for hardware, latency, and energy.

Hybrid systems could use RSS for discovery, coarse localization, or anchor selection, activating CS or direction measurements when accuracy gains justify acquisition cost. Fusion should account for uncertainty and correlated propagation errors. Inertial sensing and maps can maintain continuity between radio updates; adaptive scheduling can follow motion and uncertainty. Scalability evaluation should measure endpoint energy, processing delays, and supported devices under actual measurement schedules.

Reproducible benchmarks should document acquisition, ground truth, preprocessing, and model selection and use independent training and test sessions, heterogeneous devices, changing occupancy, and missing anchors. Overlapping temporal windows must remain in one data partition; online methods may use only observations available at estimation time. Report availability alongside distinct mean, median, root-mean-square, and percentile errors. Separate radio coverage from the evaluated positioning region (Table 13). Accuracy, latency, privacy, energy, and maintenance should guide application-specific choices between RSS and richer Bluetooth observables.

## 11. Conclusions

This review covers BLE RSS positioning from 2013 through the search cutoff of 19 September 2026, distinguishing proximity-based, model-based, and fingerprinting methods. It synthesizes reported evidence on preprocessing, sensor fusion, datasets, applications, and cross-technology comparisons.

BLE enables low-power beacons and flexible placement in GNSS-denied environments, but costs depend on scanning, battery replacement, calibration, and radio-map maintenance. RSS at 2.4 GHz remains sensitive to multipath, shadowing, body absorption, and device bias, requiring trade-offs between acquisition effort, latency, and robustness.

Windowed aggregation can reduce short-term fluctuations, typically at the cost of latency. Channel-aware methods can exploit frequency-dependent information when receivers expose channel identifiers. Fusion, including PDR, can improve continuity and control drift. Public datasets support diverse evaluations, subject to acquisition and access limits; applications include security, healthcare, navigation, and monitoring. These examples establish neither prevalence nor universal accuracy rankings. Method selection should reflect acquisition constraints, deployment conditions, and independent evaluation.

## Figures and Tables

**Figure 1:**
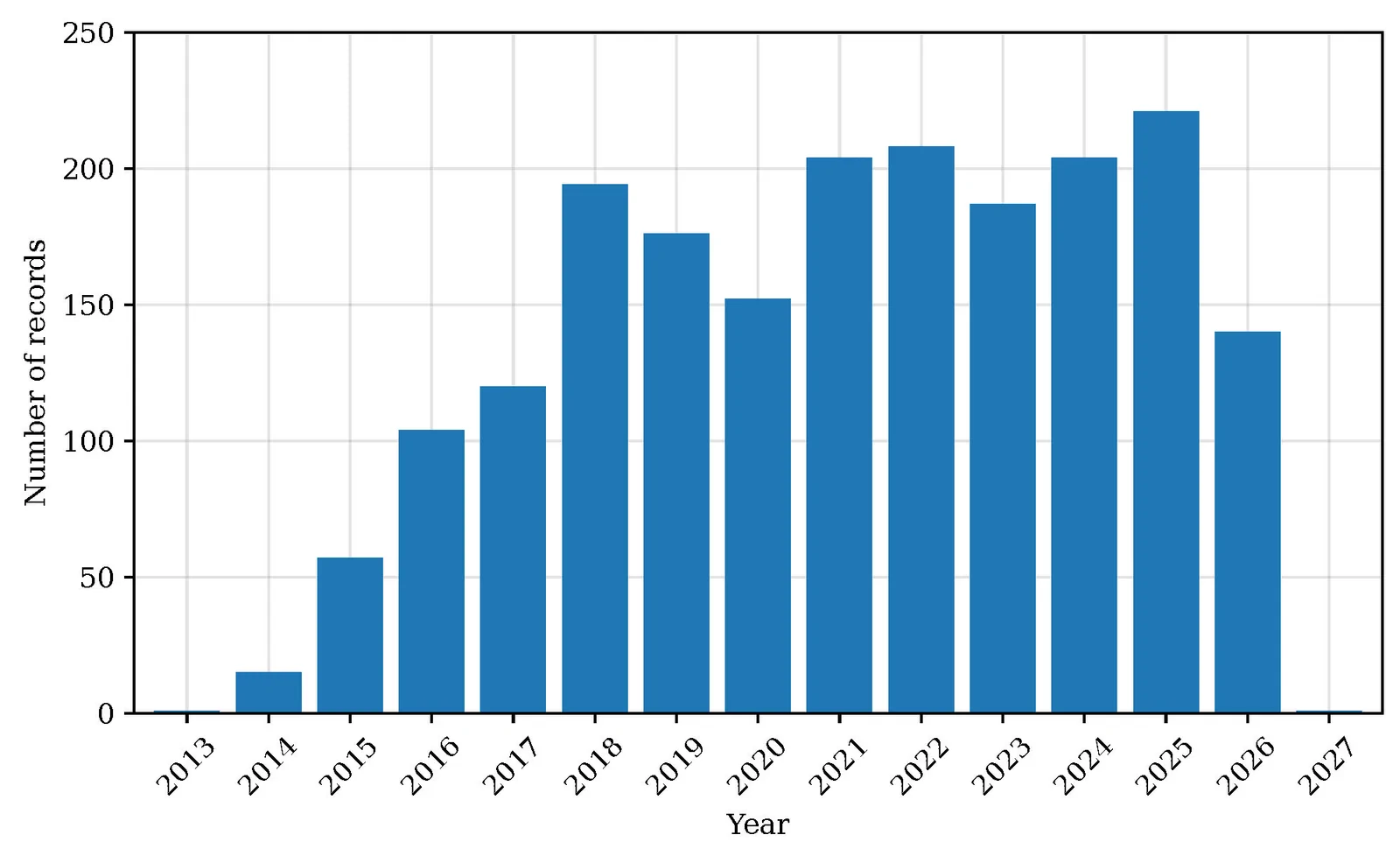
Yearly distribution of the 2136 unique database records after deduplication and before final selection. The 2026 count is partial through 19 September 2026. One Web of Science record present in the 19 September 2026 export is indexed with a 2027 issue year and is retained in the retrieval distribution.

**Figure 2:**
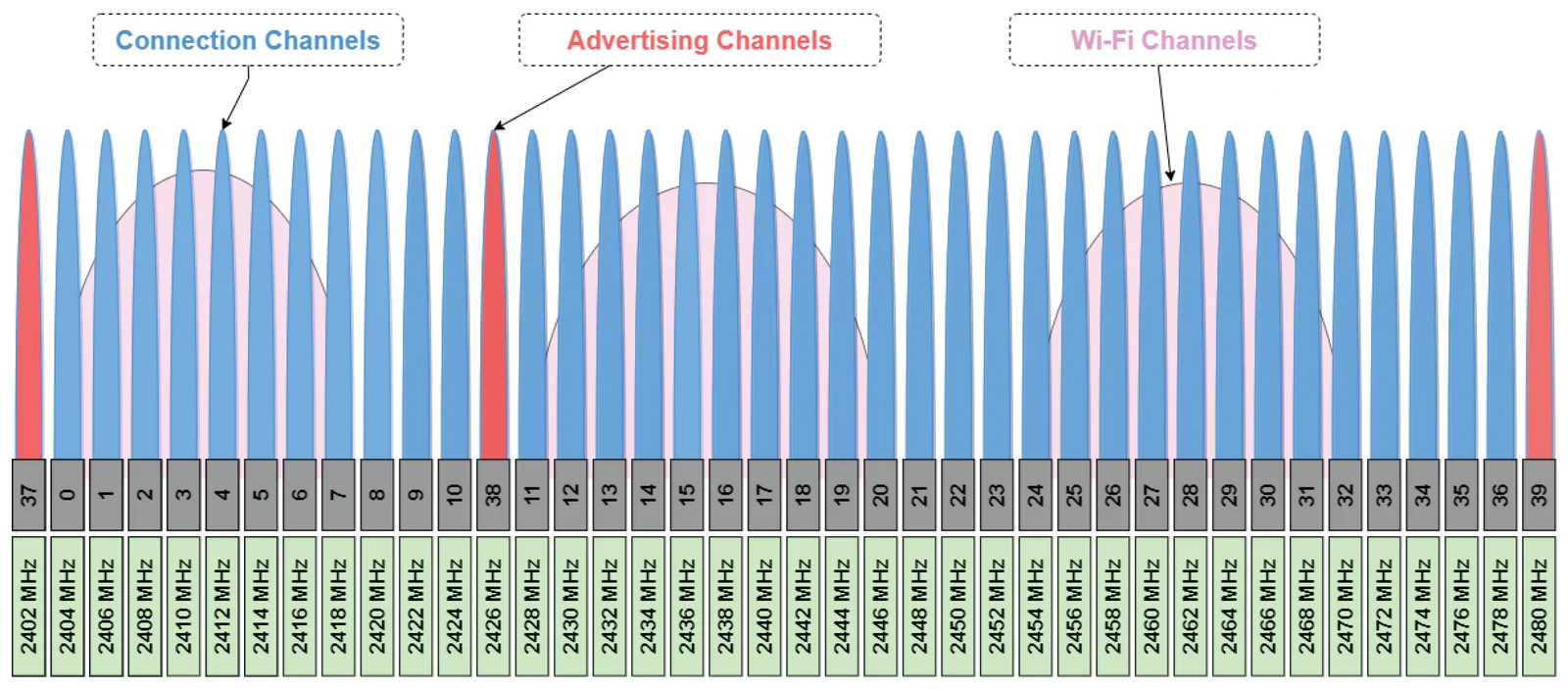
BLE channel distribution. Channels 37–39 are the primary advertising channels; channels 0–36, labeled connection channels in the diagram, also carry secondary advertising packets with Extended Advertising [12].

**Table 1:** Records retrieved from IEEE Xplore, Web of Science, and Scopus before and after deduplication. Counts include all exported results and precede relevance screening.

Source	All Results	Conferences	Journals
IEEE Xplore	1655	1264	391
Web of Science	723	380	343
Scopus	978	593	385
Total before deduplication	3356	2237	1119
Records after deduplication	2136	1444	692

**Table 2:** RSS processing and recursive state estimation for BLE positioning. Filters may act on RSS, ranges, or navigation states, as distinguished in the text.

Filter Type	Comments	References
Basic statistical aggregation	Windowed mean, median, maximum, mode, majority voting, or percentiles. The mean smooths noise but is outlier-sensitive; the median is more robust. The maximum or 90th percentile can offset fading but is peak-sensitive. Larger windows improve stability at higher latency.	[13,14,16,17,30,31,32,33,34,35,36,37,38,39,40,41,42,43,44,45,46,47,48,49,50]
Gaussian and outlier-based methods	Outlier rejection with empirical thresholds (kσ rules or statistical bounds), optionally combined with distribution modeling or smoothing. Performance depends on parameters and environment.	[18,32,50,51,52,53,54,55,56,57,58,59,60,61]
Temporal (sequential) filtering	Smooths raw or aggregated measurements to reduce threshold jitter and rapid state flips. Window length trades smoothness against latency; EWMA responsiveness depends on α. Low-pass and Butterworth filters attenuate high-frequency variation, with delay set by cutoff frequency and order.	[13,19,20,21,25,39,47,62,63,64,65,66,67,68,69,70,71,72,73,74,75,76,77,78,79,80,81,82,83]
Model-based recursive filtering (state-dependent)	Prediction–update with state and noise models. RSS-state filtering is preprocessing; range or navigation-state filtering occurs later. Particle filters represent nonlinear/non-Gaussian distributions at higher cost; performance depends on the model and particle count. References cover measurement processing and navigation-state estimation.	[18,25,26,84,85,86,87,88,89,90,91,92,93,94,95,96,97,98,99,100,101,102,103,104,105,106,107]
Channel-aware filtering	Processes RSS by channel when identifiers are available: primary advertising uses 37, 38, and 39; auxiliary packets may use secondary channels. Pooling and equal-channel weighting represent different sampling distributions. Fusion should consider offsets, aggregate uncertainty, missing samples, and correlations; separate-channel features may retain useful information.	[27,108,109]
Hybrid and adaptive strategies	Combines stages (e.g., outlier rejection, MA/EWMA, Kalman filtering), quality control, and adaptive parameters.	[16,28,35,50,75,110,111,112,113,114,115,116,117,118,119,120,121,122,123,124,125,126,127,128,129,130,131,132]

**Table 3:** BLE proximity and coarse-localization methods. Algorithm families, robustness mechanisms, and use cases overlap.

Category	Representative Works
Presence-based detection/zone entry–exit events	[135,136,137,138,139,177,178,179,180]
Highest-RSS criterion (cell-of-origin/nearest-beacon assignment)	[35,141,143,144,163,181,182,183,184,185,186]
Shortest-distance criterion (RSSI–distance/path-loss models)	[134,155,156,157,187,188,189,190,191]
Threshold-based and discretized proximity classification	[126,148,149,150,153,158,192,193]
Temporal aggregation, filtering, and learning-based robustness	[20,39,52,114,154,185,194,195,196,197]
Environmental and deployment impact	[73,120,133,167,169,198]
Hybrid or application-specific uses	[75,76,142,151,160,161,199,200,201,202,203]
Contact tracing	[174,175,176,204,205,206,207,208,209,210,211]
Learned RSS proximity/range estimation	[212]
Weighted-centroid localization (WCL/PWCL)	[213]

*Supporting sources*: Poster abstracts [214,215], the NIST report [166], and surveys [216,217] are contextual and excluded from these classifications and core counts. Fingerprint-based zone classification [218] appears in Section 5.3.

**Table 4:** BLE RSS proximity and room-classification results. Classification rates, coordinate errors and ranging-tolerance success rates are distinct metrics; SVM classification differs from simple proximity rules.

Work	Scenario/Setup	Proximity Criteria	Reported Results
[135]	Indoor/semi-outdoor/outdoor context switching	BLE RSS environment detection switches GPS/Wi-Fi on/off to save energy	Indoor/outdoor detection accuracy 96.2%; semi-outdoor localization average error 2.18 m (vs. 21.53 m GPS).
[38]	Patient-location classification in an emergency department	Mobile-app location classification using iBeacon RSS	Classification accuracy 97.22% (95% CI: 95.90% to 98.55%).
[39]	Room-level occupancy detection	SVM trained on RSS features for room-level classification	Room-classification accuracy: approximately 94%; the cited 84% proximity result is from an earlier study.
[147]	Room/zone assignment under different beacon layouts	Highest-RSS nearest-beacon coordinates; minimal calibration and fast response	Average accuracy of approximately 72% in layouts A/B and 53.33% in layout C.
[193]	Hospital corridor/zone-level tracking (symbolic positioning)	RSS thresholding and mapped-zone intersection; Channel 39, a −80 dBm reception threshold, and 100 ms tag transmissions	Exact-zone accuracy: 65.1% (989/1520 observations); exact-or-adjacent-zone agreement: 90.5% (1375/1520 observations).
[35]	BLE-tag localization with repeaters and a fusion server	Hybrid RSS fingerprinting and cell-of-origin (CoO) positioning	Reported positioning accuracy 1.2 m to 1.4 m for the hybrid system.
[199]	Industrial automated guided vehicle (AGV) guidance near rotation sub-path	Two beacons bound the rotation area; proximity within 50 cm triggers slowdown	At 50 cm, 80.2% of 3000 distance estimates were within 45 cm to 55 cm (19.8% outside the ±10% tolerance).

**Table 5:** RSS-to-distance, RSS-field, and related approaches. Range prediction, RSS-at-position prediction and joint localization/mapping have distinct outputs; categories may overlap.

Model Type	Representative Works
Standard LDPL/log-normal shadowing model (LNSM)	[17,18,28,30,32,34,36,41,44,45,51,62,66,87,89,91,95,104,113,117,147,220,221,222,223,241,242,243,244,245,246,247,248,249,250,251,252,253,254,255,256,257,258,259,260,261,262,263,264]
Polynomial regression models (RSS → *d*) and parametric residual corrections	[52,223,225,265]
Piecewise/segmented path-loss models (multi-slope or regime-based)	[32,56,74,98,236,266,267]
Extended models with structural, wall, floor, or occlusion factors (including discrete wall-count terms)	[51,135,253,268]
Sinusoidal, two-ray, ray-tracing, or physics/simulation-informed models	[31,110,231,269,270]
Channel-aware/frequency-dependent models (per-channel calibration or correction)	[63,118,226,237,261,262,271,272,273]
Noise-aware and probabilistic observation models (non-Gaussian, distance-dependent variance)	[95,198,274]
Learned RSS-to-distance or proximity mappings	[212,275]
RSS-field/radio-map prediction and calibration (including Gaussian Process Regression (GPR))	[276,277,278,279]
Joint radio-based localization and mapping	[280]
Related pipelines: particle-distribution optimization, RSS-to-distance intervals, or clustering with Bayesian estimation	[119,235,238]
Adaptive or self-calibrating RSS-to-distance models	[219,281]

**Table 6:** BLE range-based solvers and robustness mechanisms. Solver families, anchor selection, and aggregation overlap.

Class	Core Idea/Typical Solver	References
Spherical lateration (closed-form trilateration)	Maps RSSI to *d*; solves three-circle intersections or equivalent algebra for (x,y). Sensitive to multipath and geometry.	[32,87,96,110,147,247,254,267,298,299,300,301]
Spherical lateration (linearized LS multilateration)	Solves the reference-subtracted linearized range system using LS/WLS. With *m* anchors in *d* dimensions, it is overdetermined when m−1>d.	[17,236,252,302,303,304,305]
Weighted LS/link-quality weighting (WLS)	WLS emphasizes short/reliable links using distance decisions and channel-dependent weights to limit outlier impact.	[52,90,241,271,285,289,290,306,307]
Maximum-likelihood positioning	Estimates position by maximizing a measurement likelihood based on BLE RSS-derived ranges; used for UAV landing-station localization.	[308]
Nonlinear least squares (NLLS)	Minimizes nonlinear range residuals. Study-specific solvers include iterative least squares and constrained sequential least-squares programming (SLSQP), which are distinct algorithms.	[25,243,261,309]
Centroid-family (weighted centroid/AWC)	Avoids/softens range inversion via an advanced weighted centroid, sometimes initialized by LS; robust but biased toward anchors.	[36,78,291,310]
Feasible-region lateration (annuli, min–max, bounding box)	Uses rings, feasible-region intersections, or min–max/bounded boxes over the top-*k* anchors for robustness to biased ranges.	[79,80,119,123]
Robust consensus over triplets	Combines trilateration candidates using robust statistics/weighted consensus; requires enough uncontaminated, well-conditioned candidates.	[55,284]
Geometric consistency fixes (non-intersecting circles)	Resolves non-intersecting circles through proportional radius correction, reliable-constraint projection, or alternatives such as bisectors.	[74,89,98,113,293]
Anchor selection (signal quality and/or geometry)	Selects reliable anchors, optionally assessing angular diversity or conditioning. Signal-strength/distance ranking alone does not guarantee better geometry.	[25,49,84,236,287,294]

*Scope*: Differential-RSS range ratios differ from metric range differences. The 2011 reference [282] is background, excluded from primary BLE counts. Baseline solvers must not be attributed to proposed methods.

**Table 7:** Model-based BLE RSS positioning results. Labeled hybrid outcomes are not standalone BLE-only accuracy bounds.

Work	RSS→Distance/Solver	Comments	Results
[108]	Calibrated path-loss RSS→distance + channel-aware weighted trilateration + Kalman filtering	Channel diversity and weighting; Kalman filtering stabilizes static and moving estimates	Moving-device 90th-percentile error: 1.82 m in a 54 m^2^ room and 4.6 m in a 290 m^2^ room.
[247]	Path-loss RSS→distance + trilateration (circle intersections)	Two-environment comparison with Wi-Fi, Zigbee, and LoRaWAN using consistent processing	BLE mean error 0.661 m (Env. 1) and 0.844 m (Env. 2); overall BLE mean error: 0.753 m.
[254]	Log-distance RSS→distance + trilateration (3 anchors; circle intersections)	Multi-technology IoT comparison; multipath/interference produce classroom–corridor performance differences	BLE average error 0.884 m overall; the corridor error increases to 1.2725 m.
[288]	RSS→distance + trilateration (BLE refinement) in a hybrid Wi-Fi + BLE system	BLE trilateration refines the Wi-Fi fingerprinting estimate as the user approaches	BLE trilateration: 90% of estimates within 1.21 m.
[17]	Path-loss RSS→distance + least-squares lateration + EKF/PDR fusion	Graph-estimated beacon positions; results include EKF/PDR fusion	Mean error 3.25 m (dense) and 4.69 m (sparse) with estimated beacon positions.
[77]	RSS→distance + lateration with a fitted range scale (≥4 receivers) + temporal smoothing	Six locations in a 5 m × 7 m room; 30 s smoothing window and 1 s tag transmissions	Mean smoothed-position deviations from ground truth: 0.541 m to 1.215 m across six locations; five below 1 m.
[274]	RSS→distance + Rao–Blackwellised particle filtering	Simulation with four- and nine-beacon grids; additive RSS noise with standard deviation 0.2 dB to 5 dB	Particle filtering bounds simulated position error along tested paths; odometry error accumulates.
[123]	BLE localization with mobile-cloud power adaptation	Server-controlled device modes reduce interference and energy consumption	Reported mean positioning error below 1.55 m; 98% accuracy for area localization within 3 m.
[49]	RSS-based multipoint positioning + smartphone inertial measurement unit (IMU)/PDR + Kalman-filter fusion	Pedestrians walking with swinging arms; BLE fixes correct accumulated PDR error	Reduced positioning error and delay with inertial fusion.
[311]	BLE-derived ranges + dead reckoning + crowdsourced contact constraints	Seven fixed beacons over 40 m × 50 m; particle filtering and crowdsourced inference use other participants as virtual beacons	Mean errors of 2 m to 4 m across tested settings; localization latency below 1 s in all tested cases with 1, 4, 7, or 10 users.
[101]	Per-beacon RSS calibration + modified Kalman filtering + trilateration	Six equidistant beacons; a mobile robot tested in three environments (E1–E3)	Reported mean position errors: 26.67 cm (E1), 37.52 cm (E2), and 79.12 cm (E3).

**Table 8:** BLE RSS fingerprinting approaches. Algorithm families differ from orthogonal channel, maintenance, and deployment attributes; multiple tags are allowed.

Category	Description/Typical Techniques	Refs.
**Deterministic (*k*-NN/W*k*-NN)**	Euclidean/Manhattan matching; inverse-distance weighting (W*k*-NN). Simple, robust baseline sensitive to transmit power, beacon density, user/device orientation, and measurement windows/aggregation.	[57,171,314,315,316,317,318,319,320,321,322,323,324,325,326,327,328,329,330,331,332,333,334,335,336,337,338,339,340,341,342]
**Hybrid/reference-node RSS matching**	Calibration-free single-anchor localization using ESPAR antenna patterns. RSS vectors measured across antenna configurations are matched to auxiliary reference-node RSS vectors using Euclidean distance, followed by W*k*-NN position estimation. Unlike conventional fingerprinting, this approach does not require a surveyed position-indexed radio map.	[343]
**Kernel-based matching**	Beacon selection and nonlinear kernel similarities between online RSS and reference fingerprints, distinct from Euclidean/Manhattan nearest-neighbor baselines.	[344]
**Probabilistic**	Cell/position distributions via Gaussian kernels, likelihood maps, histogram fingerprints, or Bayesian inference. Often improves robustness to RSS variation or interpolated maps.	[14,276,329,337,345,346]
**Channel-aware**	Separate BLE channels (37, 38, and 39), or virtual beacons, preserve frequency-dependent information. Gains over aggregated RSS depend on experiment and metric; no universal percentage applies.	[34,52,63,347,348,349,350,351]
**Template/structure-aware**	Eight-neighborhood template matching against spatial RP neighborhoods reduces anomalous-fingerprint effects.	[352]
**Classical ML**	Room/grid classification or coordinate regression with support-vector machines, random forests, linear discriminant analysis (LDA), decision trees, or Naive Bayes. These tasks require distinct metrics.	[53,94,218,316,331,347,353,354,355,356,357,358,359,360,361,362,363,364,365,366,367,368,369,370,371,372,373]
**Physics-informed/soft-computing learning**	Semi-supervised genetic fuzzy localization; neural-network and *k*-NN baselines are distinct from the proposed model.	[374]
**Deep learning**	Autoencoders/denoising autoencoders; Convolutional Neural Network (CNN)/ANN regression; semi-supervised learning; GANs/synthetic augmentation. Often improves robustness; augmentation or crowdsourcing can reduce survey costs.	[125,230,303,375,376,377,378,379,380,381,382,383,384,385,386,387,388,389]
**Radio-map maintenance/dynamics**	Adapts to environmental changes with less calibration: radial basis functions (RBFs), KRR/GPR interpolation or densification, feedback fingerprints, and online updates.	[14,37,162,330,345,390,391,392,393]
**Deployment factors**	Performance depends on RP density, beacon count, transmit power, user orientation, and device heterogeneity. Strategies include per-orientation/direction-aware maps, cross-device handling, and grid/bandwidth trade-offs.	[19,35,48,115,171,322,324,325,326,327,329,330,331,332,354,361,362,363,392,394,395,396,397,398,399]

*Scope*: Room-level RSS classification also appears in [400]. The configuration dataset [401] is data, not an additional primary journal/conference study.

**Table 9:** BLE RSS fingerprinting and labeled hybrid results. Mean, RMSE, percentiles, and classification metrics are not interchangeable.

Work	Fingerprinting Approach	Comments	Results
[352]	Fingerprinting (ENTM)	Deterministic ENTM versus *k*-NN/W*k*-NN; percentile/RMSE improvements	RMSE 1.0 m, compared with 1.2 m for *k*-NN/W*k*-NN (16.7% reduction); 80th-percentile error 1.1 m, compared with 1.4 m (21.4% reduction).
[390]	Fingerprinting with dynamic updates	Radio-map updates under obstacles; compares the initial map, incremental feedback and interpolation	With obstacles: mean error 1.16 m with 14 feedback points, versus 1.58 m for interpolation with 14 points and 2.38 m for the initial map. The proposed method with 8 feedback points gives 1.98 m.
[248]	Hybrid BLE/inertial/magnetic localization (iBILL)	BLE clusters correct inertial/magnetic particle-filter drift; library evaluation	90th-percentile errors: 3.5 m for iBILL, 12 m for Magicol, and 17 m for dead reckoning.
[317]	*k*-NN/W*k*-NN fingerprint classification	Compares fingerprint-matching metrics using a 2 m training-grid spacing	Reported position error of approximately 1.8 m.
[314]	W*k*-NN fingerprinting (protocol/channel study)	Protocol, channel, and orientation effects on mean and 90th-percentile precision	Mean error 1.1 m to 1.5 m; 90th-percentile error 1.5 m to 2.5 m (best depends on protocol/channel/orientation).
[14]	RSS fingerprinting with different beacon densities	Comparison of high- and low-density BLE deployments	95th-percentile error: below 2.6 m (high-density BLE) and 4.8 m (low-density BLE).
[400]	BLE RSS location classification	Beacons installed in and around a room	Correct-location estimation rate above 95%; complete in-room/out-of-room separation in the reported experiment.
[355]	Room-level fingerprinting (*k*-NN/DT)	Comparison of RSS fingerprint room classifiers	Room classification accuracy 88.79%.
[147]	Standard fingerprinting baseline	Comparison baseline sensitive to environmental change and recalibration demands	Accuracy around 61%; degrades with environmental changes and requires frequent recalibration.
[17]	Walking-survey fingerprinting + W*k*-NN + Gaussian regression + EKF/PDR fusion	Walking-derived radio map; results include EKF/PDR fusion	Mean positioning errors 2.78 m (dense) and 4.11 m (sparse).
[397]	RSS–distance calibration + weighted centroid	Per-beacon RSS–distance calibration and inverse-distance-weighted coordinates; the source calls this calibration “fingerprinting”	Reported error < 1.5 m about 90% of the time; source-reported precision improvement of 13.2% over triangulation for distances up to 10 m.
[391]	Sparse walking survey + PDR path + GPR-inferred radio map	Sparse-walk GPR radio map; the PDR trajectory supports mapping/coverage with fewer samples	Average localization error 2.129 m.
[416]	BLE RSS room classification with acceleration fusion	Supervised localization estimates room dwell times and monitors daily routines	Localization accuracy above 96% with BLE RSS/acceleration fusion.
[125]	Deep-learning fingerprinting (classification/regression)	ANN feature learning/regression versus trilateration and prior ANN baselines	Regression mean error 1.9 m, outperforming trilateration (3.7 m) and prior ANN baselines (3.1 m).
[417]	PF fusion of foot-mounted IMU, BLE, Ultra-Wideband (UWB) and map matching	Integrated navigation evaluated in 10 walks along an 80 m indoor track, including stairs and running	Mean particle error below 3.1 m for 95% of the time; full-system filter completion success: 90%.
[356]	DT room classification and BLE/power-meter occupancy fusion	BLE-only localization/occupancy versus feature-level BLE RSS/aggregated-power fusion	BLE-only participant $j_{5}$: localization kappa 0.969 and own-office occupancy F-measure 97.5%. BLE/power fusion: F-measure 92.22% to 98.97% across five participants.
[322]	W*k*-NN fingerprinting + grid-resolution study	Grid-resolution/error trade-off with 12 beacons	RMSE 1.70 m for a 2 m grid compared with RMSE 1.54 m for a 1 m grid; RMSE > 5 m for 9 m to 10 m grids.
[218]	Fingerprint-based zone classification	Four 2 m × 2 m zones in a 25 m^2^ test area; zone dimensions are not coordinate-error statistics	70.2% correct zone decisions.

**Table 11:** Selected BLE RSS datasets and contextual multimodal positioning benchmarks.

Paper	Data	Purpose	Data Type	Short Description	Size/Duration	Tx/Rx	Infrastructure	Configuration	Auxiliary Data/Analysis Context
[30]	[458]	Animal monitoring/positioning	CSV/log files; experiment archives	Dairy-cow BLE collar localization; synchronized barn video ground truth.	9.8 m × 42 m; model-grid points: 235 barn, 95 waiting-yard; multi-day experiments: 35 and 19 cows.	Whole barn: 12 tags/10 Rx; waiting yard: 7 Rx	Fixed Raspberry Pi receivers; cow-worn BLE collars.	RuuviTags: 5 Hz advertising; Raspberry Pi receivers: RSS and 25 Hz acceleration.	Triaxial acceleration, RSS timestamps, video, feeding/milking annotations.
[394]	[459]	Office/grid positioning benchmark	CSV/README in ZIP	Static survey, walking-path localization, four social-interaction scenarios with two or three people.	About 185 m^2^; 277 unique grid points; 2598 labeled position records across three Tx-power campaigns.	Self: 8 Tx/1–3 Rx; remote: 1–3 Tx/8 Rx	Fixed room/ corridor BLE anchors; actors carry smartphones and wearable BLE tags.	Fixed-anchor/mobile-tag configurations; Tx powers 3, −6, and −18 dBm.	iBeacon packets, RSS timestamps, smartphones, Raspberry Pi anchors, Wi-Fi/Ethernet, MongoDB.
[460]	[461]	School indoor localization	CSV	Classroom/hall BLE RSSI from mobile devices and fixed anchors.	720 m^2^; 150 test points; 15,000 reported 1 s averaged samples.	11 mobile BLE Tx/15 fixed anchor Rx	Ceiling/wall anchors; mobile BLE devices at labeled test points.	Mobile nodes: 100 ms advertising; anchors: 1 s RSSI averages at labeled positions.	Device ID, point label, X/Y, room ID, 900 MHz gateway, MQTT protocol/Mosquitto broker, Ethernet.
[462]	[463]	Residential room-level behavior	.dat files in CSV format	Wearable BLE RSSI/acceleration with detailed residential location annotations.	4 homes; about 14 h; floor tags spaced about 1 m; 8–11 access points per home.	1 wearable Tx/8–11 Raspberry Pi Rx	Fixed Raspberry Pi access points; participant’s wrist-worn BLE device.	Wearable: 25 Hz acceleration, five samples per advertising packet at 5 Hz; receivers timestamp packets and RSSI.	Acceleration, battery, packet sequence; camera-derived location/activity annotations, fiducial-tag coordinates, annotation code.
[464]	[465]	IPIN 2018 Track 3 benchmark	GetSensorData logs; CSV/MAT ground truth, maps and scripts	Multi-floor shopping-mall smartphone data for off-site positioning.	14,000 m^2^ mall; 9000 m^2^ surveyed; 3 floors; 180 waypoints.	Existing BLE/Wi-Fi signals/smartphone Rx	Existing mall BLE/Wi-Fi; pedestrian-carried smartphone.	GetSensorData training, validation and blind-evaluation trajectories.	Wi-Fi RSS, IMU, GNSS, pressure, light, sound; evaluation scripts, raster/vector maps.
—	[466]	Room-level classification	CSV/log files	Continuous BLE scans: five beacons in four houses.	4 houses; 5 beacons per deployment.	5 fixed Tx/mobile scanner(s)	Fixed iBKS 105 beacons; labeled mobile-receiver room trajectories.	Continuous scans: anonymized MAC, timestamp, RSSI, room ground truth.	BLE RSSI time series and symbolic room labels.
[452]	[467]	Clinical tracking/occupancy	Labeled PNG RSSI images in a public GitHub repository	Real-time deep-learning BLE tag localization in a radiation-oncology clinic.	21 zones; 10 tags; 4 min per zone; 50,400 grayscale RSSI images.	10 tag Tx/21 fixed Raspberry Pi Rx	Exam-room/hallway Raspberry Pi nodes; researcher wears waist/chest tag.	RadBeacon Dot: 10 Hz advertising; 1 s RSSI averages converted to normalized images.	Released 34 × 15 RSSI images with tag/zone labels; public repository provides classification images.
[468]	[469]	IPIN 2019 Track 3 contextual benchmark	GetSensorData logs; CSV/MAT ground truth, maps and scripts	Research-campus multimodal smartphone positioning data; methodological context.	About 6000 m^2^ indoors plus 1000 m^2^ outdoors; 3 floors; evaluation log over 20 min.	Existing signals/Samsung Galaxy A5 receiver	Existing wireless infrastructure; dedicated BLE deployment unspecified.	GetSensorData: 50 training, 9 validation, 1 blind-evaluation log.	Wi-Fi RSS, accelerometer, gyroscope, magnetometer, pressure, light, proximity, AHRS, GNSS/network, and sound.
[470]	[471]	IPIN 2021 Track 3 benchmark	GetSensorData logs; CSV ground truth, maps and scripts	Smartphone indoor, outdoor and floor-transition trajectories.	University of Extremadura: external car park, ground floor and basement; over 30 BLE beacons in part of the site; 3 scoring trials.	Over 30 BLE Tx in part of the site/smartphone Rx	Partial BLE coverage plus existing Wi-Fi; pedestrian-carried smartphone.	GetSensorData/EvaalAPI: training, validation and three blind-evaluation logs.	Wi-Fi/BLE RSS, IMU, barometer, GNSS, floor plans, parsers, and evaluation code.
[472]	[473]	Mixed indoor/outdoor fingerprinting benchmark	ZIP: CSV measurements and MATLAB scripts	Multi-slot BLE RSS for smartphone positioning in campus buildings/outdoor areas.	Deployments 1/2/3: 1000/1800/5800 m^2^; 173/230/150 RPs; 744,626/869,335/114,775 scalar RSSI values, excluding −200 missing-value entries.	30 BLE Tx with 6 slots each/3, 3 and 2 smartphones in deployments 1, 2 and 3	iBKS Plus in Physics/Mathematics buildings/outdoors; user-carried smartphone.	Beacons broadcast multiple iBeacon/Eddystone slots; static samples at labeled RPs.	RSSI timestamps, X/Y, zones, beacon/slot IDs, Android low-latency scanning, training/test indices, and supporting MATLAB scripts.
[247]	[474]	Cross-technology IoT localization benchmark	Plain-text RSSI (authors’ GitHub/IEEE DataPort)	BLE/Wi-Fi/Zigbee/LoRaWAN RSSI for trilateration in two indoor office environments.	2 environments; 9 RSSI files per technology/environment; 72 radio files, including 18 BLE files.	3 BLE beacons/Raspberry Pi receiver	Gimbal Series 10 BLE beacons, Raspberry Pi Rx; three-transmitter setups for other radios.	Right-angle Tx triangle, equal legs of 1, 3 or 5 m; three Rx positions per geometry.	Tx IDs, RSSI; cross-technology trilateration, study-specific power measurements.
[66]	[475]	Gait-speed estimation	CSV	Corridor BLE RSSI during constant-speed walking.	14 m corridor; 382 walks by 13 actors.	20 beacons (19 valid)/4 smartwatches	Ceiling iBKS beacons; users wear Sony Smartwatch 3 on both wrists.	Smartwatch beacon scans; synchronized ultrasonic speed ground truth.	Ultrasound, Arduino/NTP, MAC, RSSI, device, timestamp, user, direction, walk ID and speed.
[454]	[476]	Contextual outdoor fingerprinting	CSV	OutFin generic-Bluetooth/multimodal outdoor benchmark; methodological context.	4 outdoor sites; 122 RPs; 61–183 cm RP spacing; 2 smartphones.	Existing Bluetooth devices/2 smartphones	Existing outdoor wireless signals; tripod-mounted smartphones at RPs.	Samsung Galaxy S10+, Google Pixel 4 fingerprints at fixed campus RPs.	Wi-Fi, Bluetooth, cellular, GPS, magnetometer, accelerometer, gyroscope, barometer, light and ground truth.
[117]	[477]	BLE RSS variability/LOS-NLOS proximity	Zenodo CSV archive	Controlled Tampere/Bucharest BLE RSS under LOS/NLOS and interference.	Distances: 1–3 m; multiple fixed indoor snapshots.	1 Raspberry Pi 4 Tx/1 Raspberry Pi 4 Rx	Static devices; known distances/orientations.	Controlled distance, orientation, obstacles, doors/walls, body shadowing, Wi-Fi coexistence.	Advertising channels 37/38/39, timestamps, path-loss analysis, environment labels.
[279]	[478]	Robot-assisted radio-map calibration	ZIP: raw/processed data, LiDAR scans, and Python loader (dataset release 1.0)	Mobile-robot BLE RSS fingerprints/trajectories in a furnished apartment.	Furnished apartment; measurements/LiDAR at 4104 points.	1 mobile BLE tag/6 fixed BLE anchors	Fixed anchors; robot- or pedestrian-carried tag.	Robot-acquired radio map; robot/person test trajectories.	LiDAR, odometry, GraphSLAM, Raspberry Pi, path-loss, GPR, ANN and Random Forest.
[445]	[479]	Smart-home room-level localization	TGZ/CSV recordings	WatchBLoc recordings during natural activities in a demo home and real flat.	Demo home: 6 rooms; real flat: 4–6 semantic rooms; 20 labeled recordings, including alternative room partitions of the same sessions.	3 beacon placements per room/Sony Smartwatch 3	Beacons at room-center, distant and doorway positions; participant wears smartwatch.	Smartwatch logs ambient BLE RSSI and IMU.	Accelerometer, gyroscope, magnetometer, room ground truth, HMM/probabilistic model and semantic mapping.
[312]	[480]	Large-scale BLE fingerprinting benchmark (UEx URI)	Publisher supplement: database and MATLAB material	Multi-floor smartphone fingerprints for deterministic/probabilistic method comparison.	3 floors, each approximately 80 × 35 m; 200 RPs + 80 test points; 4 orientations; 800 training + 320 test fingerprints per phone.	60 beacons with 6 slots (up to 360 signals)/5 smartphones	iBKS Plus beacons throughout building; users carry smartphones.	Labeled points, orientations and floors; 10 s offline and 2 s online scan windows.	RSSI, timestamps, beacon/RP/orientation IDs, phone model, BLE version, floor and coordinates.
[481]	[482]	Hybrid Wi-Fi/BLE robustness under layout changes	CSV and spreadsheet files in ZIP	HDLC multi-floor fingerprints under three partition-board layouts.	386.25 m^2^; 3 floors; 384 RPs on a 1 m grid; 30 samples per RP; 3 layouts.	42 BLE beacons + 17 Wi-Fi APs/1 smartphone	Sensoro beacons and D-Link routers on corridor walls; handheld Galaxy Note10 Lite.	Layout 1: no boards; Layout 2: boards on ground and second floors; Layout 3: boards on all floors.	BLE/Wi-Fi RSSI, 3D coordinates, floor/layout labels; missing signals = −110 dBm; 4 dBm, 417.5 ms.
[448]	[483]	Bluetooth 5.1 AoA/RSS benchmark	Delimited TXT time series in ZIP	Calibration, static, mobility and proximity recordings with AoA, RSS and ground truth.	110 m^2^ room (13.8 × 8 m); 119 calibration and 36 static points; 3 mobility and 4 proximity cases; 4,016,625 raw beacon-log records in v1 (two RSS values per record).	4 BLE tags/4 fixed AoA anchors	Four u-blox XPLR-AOA-1 anchors at room perimeter; tripod-mounted or body-worn tags.	50 Hz advertising; static positions, walking paths and proximity interactions.	AoA azimuth/elevation, dual-polarization RSS, channels 37/38/39, timestamps and X/Y ground truth.
[484]	[485]; Kaggle benchmark	Large-scale indoor localization competition	Zenodo sample ZIP; full smartphone-log benchmark on Kaggle	Indoor Location Competition 2.0: multi-building smartphone traces.	Full benchmark: approximately 60 GB across hundreds of buildings; cited Zenodo release: sample only.	Existing Wi-Fi/BLE where available/smartphones	Existing APs/beacons and mapped landmarks; no dedicated infrastructure.	Waypoint/landmark surveys yield training/test trajectories on floor plans.	Wi-Fi RSSI, BLE where available, IMU, geomagnetic data, maps, POIs and PDR routines.
[456]	[457]	Wi-Fi/BLE LOS/NLOS classification	CSV, MATLAB code and documentation	Labeled indoor Wi-Fi/BLE RSS for LOS/NLOS detection.	2 environments; 20 BLE beacons (10 LOS + 10 NLOS); 10 campaigns; at least 3 h per campaign.	BLE beacons and existing Wi-Fi APs/Android smartphones and tablets	Fixed iBKS Plus beacons and existing APs; mobile RSS receivers.	100 ms advertising; Tx powers from −30 to 4 dBm; continuous labeled Wi-Fi/BLE scans.	Raw RSS, timestamps, transmitter IDs, Wi-Fi band, BLE Tx power, histograms, NN/SVM/linear models.
[27]	[486]	Channel diversity and Extended Advertising	IEEE DataPort CSV	Per-channel BLE RSSI from legacy/extended advertisements across up to 40 channels.	100 m^2^ office; 105 test-grid + 19 calibration points.	1 mobile nRF52840 Tx/4 fixed nRF52840 Rx	Four corner anchors; mobile node moved over grid.	Anchors log RSSI and channel IDs on primary/secondary advertising channels.	Path-loss, weighted multilateration, Geo-N localization, channel selection and BLE 5 Extended Advertising.
[166]	[487]	COVID-19 proximity detection	NIST ZIP: README and data files	Controlled BLE RSSI for LOS/NLOS electronic contact tracing.	5-week campaign; 144,581 samples (83,024 LOS; 61,557 NLOS).	2 Raspberry Pi Zero W devices alternating Tx/Rx roles	Tripod-mounted wearable devices at measured distances.	3 min per direction; distance, orientation, people, walls and materials varied.	RSSI, timestamps, device ID, channels 37, 38, and 39, LOS/NLOS, orientation/material metadata and detector analyses.
[488]	[489]	BLE fingerprinting with improved particle filtering	CSV fingerprints in RAR	RSSI fingerprints comparing k-NN, SVM, and improved particle-filter likelihoods.	7.8 × 7.9 × 2.8 m room; 73 locations (51 RPs, 22 test points); 3 min per point.	4 ceiling beacons/Honor 8 smartphone	Fixed ceiling beacons; smartphone localization.	Uniformly distributed fingerprint points in furnished room; offline map and test points.	k-NN, SVM, particle filter, Gaussian mixtures, likelihood functions and obstacle layout.
[450]	[490]	Indoor BLE fingerprinting	CSV fingerprints and MATLAB/Octave scripts in ZIP	University library/office BLE RSS, including device and power variability.	Library: 176.02 m^2^, 2100 fingerprints, 24 beacons; office: 151.07 m^2^, 2652 fingerprints, 22 beacons.	24/22 beacons/3 phones in library, 1 phone in office	iBKS 105 beacons on bookshelves/ceiling tiles; users carry Android smartphones.	200 ms advertising; library: −12 dBm; office: −4, −12, and −20 dBm.	Timestamps, IDs, coordinates, orientation, obstacles, power levels and positioning scripts/examples.
[491]	Zenodo archive	Passive Wi-Fi/Bluetooth contact tracing and localization	ZIP/CSV and ROS 2 PGM map files (GitHub/Zenodo mirror)	Router-side Wi-Fi/BLE packet features and robot-labeled contact-tracing trajectories.	Multiple university floors; 12 train/test datasets; over 1,000,000 points; up to about 1000 positions per floor.	Phones/mobile devices as Tx/fixed custom and commercial off-the-shelf (COTS) routers as Rx	ESP32-C3/COTS routers; TurtleBot3 carries smartphone for site survey.	Autonomous robot supplies time/position labels; independent train/test sets by floor/site.	Wi-Fi RSSI/SQI/FTM-RTT, BLE Tx power, timestamps/MAC/model, LiDAR, RGB-D, ROS 2 and BiLSTM.
[492]	[453]	Separate-channel fingerprinting and antenna comparison	Semicolon-delimited TXT in ZIP (v2)	Unified/separate-channel BLE fingerprinting with monopoles and frequency-scanned leaky-wave antennas.	35 m^2^ room (7 × 5 m); 130 points on a 0.5 m grid; 94-day span; 717,418 scalar RSSI readings (358,618 FSLWA; 358,800 monopole).	1 mobile BLE Tx/4 monopole Rx + 4 BLE receivers connected to 2 dual-port FSLWAs	Four ceiling monopoles and two ceiling dual-port FSLWAs; transmitter moved over floor grid.	Day-0 calibration; tests on days 1, 8, 15, 22, 29, 51, 86 and 94; furniture tests on days 15, 22, 29, 51 and 94.	Channels 37/38/39, timestamps, receiver-port IDs; grid coordinates encoded in filenames.
[493]	[494]	Crowd sensing and coarse nightclub localization	ACM artifact ZIP: BLE packet and sensor logs	CWI-ADE2016: real-life nightclub BLE wristbands and fixed receivers.	Over 40 million BLE reception records; about 14 million sensor-data records; about 900 guests; about 500 m^2^; 2 nights.	812 Estimote devices, 109 SensorTags, and other devices/17 and then 13 Raspberry Pi Rx	Raspberry Pi receivers across rooms/dance floors; guests wear custom BLE wristbands.	Broadcast sensor payloads forwarded by receivers to a central MongoDB server.	Acceleration, temperature, timestamps, hashed IDs/payloads, receiver ID, room/crowd and packet-copy analyses.
—	[451]	Industrial BLE localization (SAL-RB)	CSV in versioned ZIP	Labeled RSSI from a node moving through an automotive testbed.	More than 20,000 observations per sniffer; 2 walking and 8 vehicle measurement sets.	1 measurement node per capture/10 sniffers; base station receives traffic.	Sniffers inside/outside testbed at AVL List, Graz.	nRF52840 hardware; 100 ms EPhESOS superframe/measurement interval.	Region labels from two photosensors; raw photosensor data and sniffer coordinates supplied.
[455]	[495]	Industrial navigation/radio interference	CSV measurements (GitHub)	Robot-mission BLE/Wi-Fi RSSI with synchronized navigation ground truth.	178.60 m^2^; four rooms and an aisle; repeated robot trajectories.	16 BLE beacons + 14 Wi-Fi ESP32 anchors/robot-mounted receiver.	Fixed radio nodes at 17 positions; MiR100-mounted receiver.	Packet records linked to robot position, orientation, velocity and mission.	Timestamps, MAC, X/Y, orientation, linear/angular velocity and trajectory labels; robot sensors support ground truth.

*Note*. Repository metadata were checked on 19 September 2026; the WNLab archive was accessible on 18 February 2026 but unavailable during the final link check. Full repository URLs and access dates are given in the references. The Zenodo sample and full Kaggle benchmark are distinct resources. Software names in the table refer to third-party dataset materials, with versions reported only when provided by the cited source.

**Table 12:** BLE positioning application domains; multiple tags are allowed. Inclusion here does not establish core methodological eligibility.

Category	Representative Works
Navigation and user assistance	[73,74,136,137,145,168,192,199,200,225,256,497,503,504,515,524,529,530,531,532,533,534,535]
Healthcare and assisted living	[38,40,62,117,122,191,205,206,208,210,216,231,241,410,427,500,501,536,537,538,539,540,541,542,543,544,545,546,547,548,549,550,551,552]
Safety, security, and emergency management	[86,88,164,251,405,505,506,507,514,553,554,555,556,557,558,559,560,561,562,563,564,565,566]
Advertising	[187,567,568]
Resource management and smart environments	[39,91,141,180,200,218,244,320,352,355,402,499,509,510,511,569,570,571,572,573,574,575,576,577,578,579,580,581,582,583]
Animal and agricultural monitoring	[30,165,513,584,585,586]
Construction, mining, and industrial	[83,251,516,517,518,519,520,521]
Education, augmented reality, and tourism	[522,523,587,588,589,590,591,592,593,594,595,596,597,598,599,600,601,602]
Crowdsensing	[508,525,526,527,528,603,604,605,606,607]

**Table 13:** BLE RSS and alternative technologies. Reported errors are study-specific; coverage and deployment considerations are qualitative. Timing- and angle-based positioning results are excluded.

Technology	Reported Error/Capability	Range/Scale	Energy, Infrastructure, and Cost Considerations
BLE RSS	Mean: 1.47–2.15 m [33]; average: 0.753 m [247].	Meters to tens of meters per beacon.	Low-power, inexpensive beacons; scanning, batteries, and map upkeep add effort. Sensitive to multipath and device differences.
Wi-Fi RSS	Mean: 3.94–4.82 m [33]; average: 0.664 m [247].	Room/building coverage.	Can reuse access points; scanning consumes energy. Calibration, device differences, and scan restrictions affect performance.
Classic Bluetooth RSS	Mean horizontal error: 5.1 m [608].	Short-range indoor links.	Cited setup: dedicated access points and radio map. Discovery delays, missed responses, and inquiry energy matter.
Zigbee RSS	Average: 0.896 m [247].	Local anchor coverage.	Low-power end devices; dedicated anchors and gateways. Smartphones usually require external hardware.
LoRa/LoRaWAN RSS	Small indoor RSS study [247]; inconsistent source values preclude an aggregate error.	Potential building/site coverage.	Low-duty-cycle nodes and dedicated gateways. Airtime, anchor geometry, and reporting rate constrain positioning.
UWB RSS	Coordinate MAE: 0.33 m (flat A), 0.35 m (flat B); long short-term memory (LSTM)+CNN, past-only 12 s window [609].	Two flats evaluated; operational link range not reported.	Dedicated tags, anchors, and training data. Energy varies; RSS access and temporal windows affect comparability.
NFC	Proximity/checkpoint identification [610].	Centimeters.	Battery-free tags powered by readers. Requires close interaction; unsuitable for continuous room-scale tracking with one reader.
Passive RFID	Room/proximity detection and contact tracing with passive UHF tags [176].	Band-dependent: near-field HF or longer-range UHF.	Battery-free tags; powered readers. Reader installation, materials, and tag orientation affect deployment.

*Note*: Different devices, geometries, and metrics preclude a common accuracy ranking. Values from [247] are multi-radio test averages; its inconsistent LoRaWAN aggregate is omitted. UWB coordinate MAE averages absolute X/Y errors, not mean Euclidean error; the temporal window is an evaluation condition. Coverage does not guarantee the listed accuracy. Energy/cost descriptions are qualitative, depending on endpoint role (advertiser, scanner, and active or passive tag), acquisition duty cycle, and reused versus new infrastructure; no common power or monetary budget is implied. HF and UHF RFID differ; active RFID is excluded.

## Data Availability

This review synthesizes previously published studies; no new experimental BLE RSS measurements were collected. The methodological classifications and extracted performance summaries are presented in the manuscript tables, with the underlying publications identified in the reference list. Table 11 and the corresponding references identify third-party datasets and their repository records. Access to these resources is governed by the respective providers. A separate publication-level screening and extraction register is not supplied with this article.

## Abbreviations

The following abbreviations are used in this manuscript:

AGV	Automated Guided Vehicle
ANN	Artificial Neural Network
AoA	Angle of Arrival
AoD	Angle of Departure
AP	Access Point
BLE	Bluetooth Low Energy
BR/EDR	Basic Rate/Enhanced Data Rate
CNN	Convolutional Neural Network
COTS	Commercial Off-the-Shelf
CS	Channel Sounding
DRL	Deep Reinforcement Learning
DT	Decision Tree
EKF	Extended Kalman Filter
ENTM	Eight-Neighborhood Template Matching
EWMA	Exponentially Weighted Moving Average
FSLWA	Frequency-Scanned Leaky-Wave Antenna
GAN	Generative Adversarial Network
GAP	Generic Access Profile
GATT	Generic Attribute Profile
GFSK	Gaussian Frequency-Shift Keying
GNSS	Global Navigation Satellite System
GPR	Gaussian Process Regression
GPS	Global Positioning System
HCI	Host Controller Interface
HMM	Hidden Markov Model
HVAC	Heating, Ventilation, and Air Conditioning
IMU	Inertial Measurement Unit
IoT	Internet of Things
IPS	Indoor Positioning System
ISM	Industrial, Scientific, and Medical
KF	Kalman Filter
*k*-NN	*k*-Nearest Neighbors
KRR	Kernel Ridge Regression
LDA	Linear Discriminant Analysis
LDPL	Log-Distance Path Loss
LNSM	Log-Normal Shadowing Model
LoS	Line-of-Sight
LS	Least Squares
LSTM	Long Short-Term Memory
MA	Moving Average
MAD	Median Absolute Deviation
MAE	Mean Absolute Error
MCPD	Multi-Carrier Phase-Difference
ML	Machine Learning
MSE	Mean Squared Error
NFC	Near-Field Communication
NLLS	Nonlinear Least Squares
NLoS	Non-Line-of-Sight
PBR	Phase-Based Ranging
PDR	Pedestrian Dead Reckoning
PF	Particle Filter
PHY	Physical Layer
RBF	Radial Basis Function
RF	Random Forest
RFID	Radio-Frequency Identification
RMSE	Root Mean Squared Error
RP	Reference Point
RPs	Reference Points
RSS	Received Signal Strength
RSSI	Received Signal Strength Indicator
RTLS	Real-Time Locating System
RTT	Round-Trip Timing
SLAM	Simultaneous Localization and Mapping
SPAD	Single-Photon Avalanche Diode
SVM	Support Vector Machine
TDoA	Time Difference of Arrival
ToF	Time of Flight
UKF	Unscented Kalman Filter
UWB	Ultra-Wideband
VAE	Variational Autoencoder
Wi-Fi	IEEE 802.11 Wireless LAN
W*k*-NN	Weighted *k*-Nearest Neighbors
WLAN	Wireless Local Area Network
WLS	Weighted Least Squares
WPAN	Wireless Personal Area Network
